# A novel APP splice variant-dependent marker system to precisely demarcate maturity in SH-SY5Y cell-derived neurons

**DOI:** 10.1038/s41598-024-63005-y

**Published:** 2024-05-27

**Authors:** D. Chanuka M. Kulatunga, Umanthi Ranaraja, Eun Young Kim, Ryoung Eun Kim, Dong Ern Kim, Kuk Bin Ji, Min Kyu Kim

**Affiliations:** 1 Laboratory of Animal Reproduction and Physiology, College of Agriculture and Life Sciences, Chungnam National University, Yuseong-gu, Daejeon, 34134 Republic of Korea; 2 MK Biotech Inc., Daejeon, Republic of Korea

**Keywords:** Neurological models, Transcription, Gene expression, Molecular neuroscience, Biological techniques, Cell biology, Neuroscience, Biomarkers

## Abstract

SH-SY5Y, a neuroblastoma cell line, can be converted into mature neuronal phenotypes, characterized by the expression of mature neuronal and neurotransmitter markers. However, the mature phenotypes described across multiple studies appear inconsistent. As this cell line expresses common neuronal markers after a simple induction, there is a high chance of misinterpreting its maturity. Therefore, sole reliance on common neuronal markers is presumably inadequate. The Alzheimer's disease (AD) central gene, amyloid precursor protein (APP), has shown contrasting transcript variant dynamics in various cell types. We differentiated SH-SY5Y cells into mature neuron-like cells using a concise protocol and observed the upregulation of total APP throughout differentiation. However, APP transcript variant-1 was upregulated only during the early to middle stages of differentiation and declined in later stages. We identified the maturity state where this post-transcriptional shift occurs, terming it "true maturity." At this stage, we observed a predominant expression of mature neuronal and cholinergic markers, along with a distinct APP variant pattern. Our findings emphasize the necessity of using a differentiation state-sensitive marker system to precisely characterize SH-SY5Y differentiation. Moreover, this study offers an APP-guided, alternative neuronal marker system to enhance the accuracy of the conventional markers.

## Introduction

Neuronal cell lines play a crucial role in neuroscience research as in vitro model systems that mimic the nervous system and its associated disorders at the molecular, physiological, and cellular levels. Neuroblastoma (NB) cell lines are more versatile than other neural cell lines because they are neuroblast-like, undifferentiated neural precursors derived from the neural crest and could differentiate into mature neuronal cell types. Among NB cell lines, such as rat PC-12, mouse neuro-2a, and human SH-SY5Y, SH-SY5Y stands out as the most widely used cell model in neurodegenerative research. This cell line, derived from a sub-cloned metastatic bone marrow biopsy of a patient with neuroblastoma^1^, is a well-established model in neurological research, including investigating the molecular mechanisms underlying numerous disorders, such as Parkinson's disease (PD) and Alzheimer's disease (AD). Moreover, the SH-SY5Y cell line can differentiate into multiple neuronal phenotypes, making it a more attractive tool for studying neurons. The SH-SY5Y cell line has been frequently used in both its undifferentiated and differentiated states^2^. Proliferating SH-SY5Y cells represent an undifferentiated state, which closely resembles that of neural progenitor cells or immature neurons. Although they have certain merits, including ease of culture and reproducibility, the validity of experiments conducted using undifferentiated neuronal cell lines is often disputed as these cell lines fail to reflect the complexity of mature neurons and their stimuli-response mechanisms^2^.

To develop a more realistic neuronal model, it must be converted to a mature neuronal phenotype by inducing differentiation. The differentiated state mimics mature neurons, thus exhibiting mature neuronal markers, such as SYP, NSE, and MAP2, and lacking glial (GFAP) and oligodendrocyte (OLIG2)-like non-neuronal markers. Consequently, the SH-SY5Y cell line is widely used as a model for Parkinson’s disease focusing on its dopaminergic phenotypic characteristics^2,3^. Moreover, SH-SY5Y cells can also be differentiated to exhibit cholinergic phenotypes^4^. The most common method for differentiating SH-SY5Y cells is the addition of all-trans-retinoic acid (ATRA), hereafter referred to as retinoic acid (RA). RA is solely involved in neural maturation^5^ and in axon and dendrite development during neuronal differentiation^6^. However, the combination of RA and the brain-derived neurotrophic factor (BDNF) induces neuronal differentiation, enhances neurite outgrowth^7^, and increases neuronal survival^8^, leading to the production of a relatively mature neuronal phenotype^8,9^.

Multiple procedures have been developed that preferentially direct neurons toward their specific matured types, primarily classified based on the mature neuron markers and the type of neurotransmitter they produce, such as dopamine and acetylcholine. However, the differentiated states of the SH-SY5Y cells described in most studies appear to be inconsistent, as detailed in Table 1. Given its neural progenitor-like nature, SH-SY5Y cells can potentially express common neuronal markers, even with slight differentiation. In this setting, using common neuronal markers alone to identify the exact differentiation state is improbable.

**Table 1:** Duration of SH-SY5Y differentiation described in each study.

Study objective	Terminal morphology appeared at (days)	Morphological data collected at, (days)	Marker screening method and time (days)	Observed until (days)	Markers expressed at the differentiated state	
					Marker positivity	Negative
PD, Dopaminergic model^3^	1	7	WB, ICC 4–10	10	NSE, NeuN, TH	
Mature neuron model^10^	3	3	WB, ICC 3	3	GAP43, β3-tubulin, FAK	-
Cholinergic neuron, AD model^11^	4	7	WB; 7, Mc. Array; 4	7	AChE, ChAT, DAT, SCL18A(VACHT), CDK5, PSEN1	-
Differentiation model^12^	6	6	QPCR, ICC 3,6	6	ACHE, NES, SOX2, SYP, SYN1, TUBB3, etc.…	-
AD model^13^	7	7	WB 7	7	BACE-1, PSEN-1	-
Differentiating method^7^	10	7	ICC 10 (or 5)	10	β-III tubulin, Tau and GAP-43	-
Ageing model^14^	14	14,28	ICC 14	28	βIII-tubulin, TH, synapsin	-
Differentiating method^15^	18	18	ICC 18	18	GAP-43, NeuN, SYN, SV2, NSE, MAP,	GFAP
PD, Dopaminergic model^16^	30	30	QPCR, WB, ICC 30	40	SYP, TUBB3, NRG1, MAPT, MAP2, TH, DRD2, DAT, NEUROD1, NEUN	-
3D culture, Cholinergic^17^	40	10, 20, 30, 40	ICC 10, 20, 30, 40	48	SYN1, PSD95, AChE, ChAT	
Our study	22	Series 1–27, 30	QPCR 1–25 (APP) 22 (other)	32	ENO2, MAP2, SYP, MAPT, VACHT, ACHE, GABBR1, APP, PSEN1, BEX1, BEX3	AIF1, GFAP, OLIG2, CSPG4

The time taken to acquire the ultimate state of differentiation, total observed duration, and expressed mature neuronal markers are indicated.

Therefore, we introduce the amyloid precursor protein (APP) transcript variant-based marker system to characterize the differentiation state of SH-SY5Y cells.

APP is a ubiquitously expressed transmembrane protein and is found abundantly in neurons. It is known as the central protein in AD and is also among the few members that cause inherited, autosomal dominant, early onset AD (EOAD). Proteolytic cleavage of APP allows for the rise of the pathogenic forms of amyloid beta (Aβ), the major component in senile plaques and one of the two major pathological signs in AD brains^18,19^. The onset of Aβ accumulation in the brain ultimately triggers AD^20^. Apart from its classical role in AD pathogenesis, the expression of APP is worth studying as a marker system in neuronal differentiation. In most tissues, the APP transcript variant 1 (also known as APP770 or isoform a) is the dominant transcript variant^21^. In the context of the brain, total APP is more abundantly expressed than in most tissues^22^ and, compared to the isoform APP770, the isoform APP695 is dominant^23^. Moreover, neurons in the human brain cortex showed a higher level of APP expression^23^, as a similar pattern is also observed in differentiated SH-SY5Y cells^24^.

The primary objective of our study is to precisely differentiate SH-SY5Y cells and observe the dynamic expression patterns of the APP and its transcript variants during the differentiation process with the aim of validating the APP transcript variant dynamics as a novel marker system to demarcate the terminal differentiation states ("true maturity") of SH-SY5Y cells.

In this study, we adopted a simple protocol to differentiate SH-SY5Y cells and focused on the expression of the APP gene and its quantifiable transcript variant dynamics in each differentiation state, along with the corresponding cell differentiation morphology. For differentiation, the cells were maintained in RA-amended DMEM/F12 throughout the experiment, and the serum in the medium was sequentially deprived until achieving a serum-free condition. We employed a series of differentiation time points for morphological and transcriptional analysis and observed an increasing trend in total APP expression throughout differentiation. We observed APP gene expression and identified a distinct point in the differentiation curve where the major shift occurs in the APP transcriptional composition, termed "true maturity". In addition, we observed a predominant expression of mature neuron and cholinergic markers, accompanied by a significant increase in total APP expression, dominated by transcript variant 3, in differentiated cells.

Our study highlights the optimal terminal differentiation conditions for the SH-SY5Y cell line, introduces APP transcript variant dynamics as a potential marker system to detect the specific differentiation states of these cells, and may potentially extend the utility of this cell line in neurobiology research.

## Results

### Differentiation of SH-SY5Y cells into mature neurons

**Figure 1:**
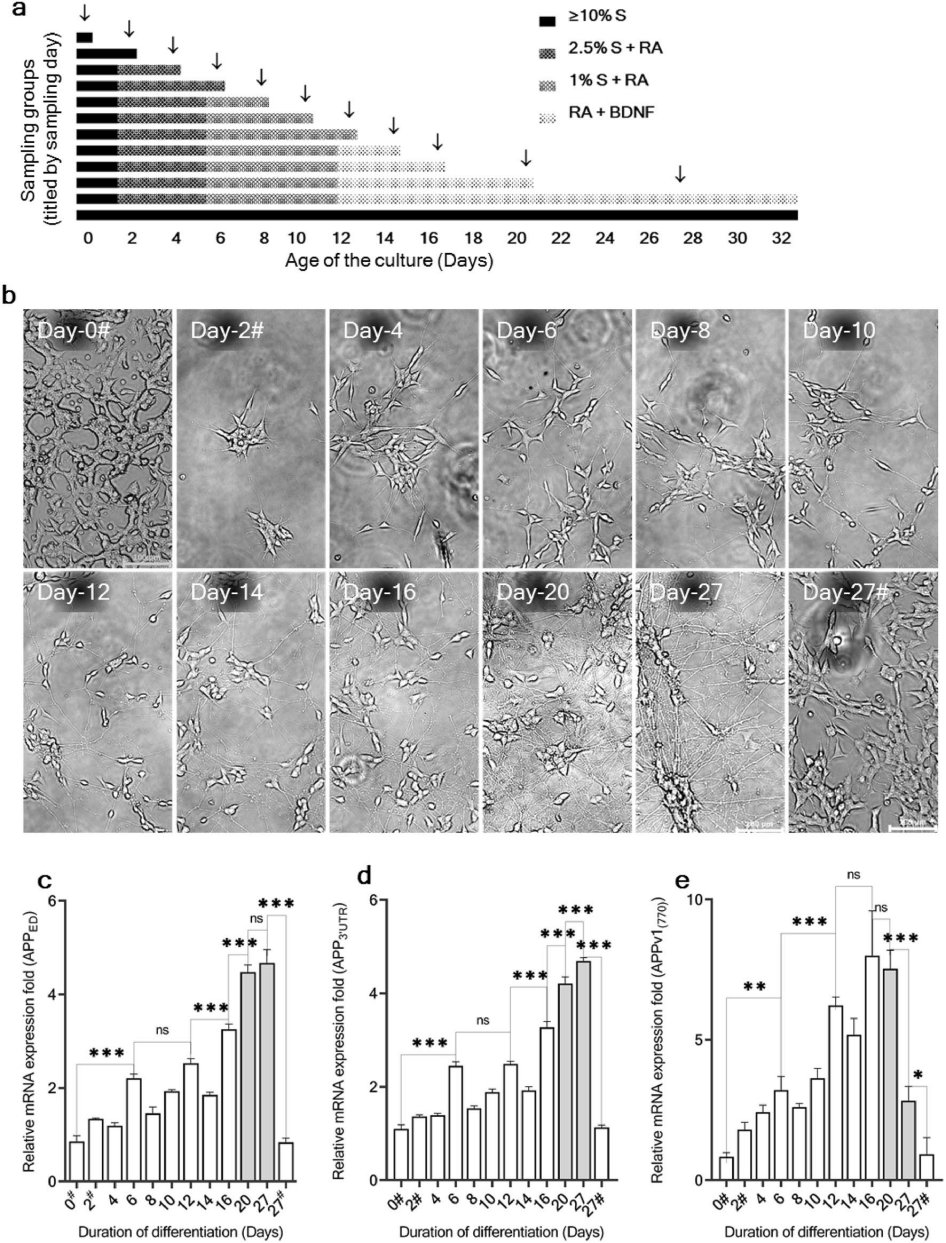
Timeline of the SH-SY5Y differentiation and the alongside APP expression. (**a**) The media composition and the sampling. The wt-SH-SY5Y cells were used to study the endogenous APP dynamics during neuronal differentiation. Each row represents each sampling group, designated based on the culture age (days) at the sampling (from day 0 to day 27). Media serum (≥ 10% S, 2.5% S, and 1% S), RA (started from day 2), and BNDF (started from the day 12) combinations were differentially shaded. The sampling groups maintained under proliferative culture conditions (≥ 10% media serum and without RA) until the day of sampling were filled in black, and the cells under differentiation conditions were shaded with different patterns. Each sampling point was indicated by an arrow. The cell morphology at each sampling point is depicted in Fig. 2b. (**b**) Representative images of the time-course morphological alteration of SH-SY5Y during differentiation. Proliferating, undifferentiated SH-SY5Y cells were maintained in 15% serum. These cells were used as the cell resource in subsequent differentiation experiments, denoted as day-0# (Control-1). A day-grown cells in 10% serum following the low dense seeding, denoted as day-2# or (Control-2). The culture passaged long-term under the proliferative condition is denoted as day-27# (Control-3). The day-4 and day-6 cells were maintained in the 2.5% serum from the day-2. The day-8, day-10, and day-12 cells were maintained in the 1% serum from the day-6. From day 12, the cells were maintained in a full serum cut-off. Images were taken using an inverted microscope (×10, scale bar; 100 µm). The APP mRNA expression dynamics throughout the differentiation by targeting the common APP extracellular domain (**c**), APP 3ʹUTR (**d**), and the 7 and 8 exons of APP770 (**e**) in QPCR. The time points where the major splice form shift is taking place were shaded. The gene GAPDH was used as the reference internal control for the normalization. The cells at proliferative conditions (≥ 10% serum) were marked with "#". The statistical significance at p < 0.05, represented as "ns": *p* > 0.05 (not significant), "*": *p* ≤ 0.05, "**": *p* ≤ 0.01, and "***":*p* ≤ 0.001. Error bars represent the mean SD (n = 3).

SH-SY5Y (wild-type) cells were successfully differentiated into a mature neuron-like phenotype. The differentiation process was closely monitored for morphological changes. Microscopic observations were made, and photographs were taken at time points parallel to the sampling (Fig. 1a). Continuous neurite extension was observed throughout the differentiation process. During regular passaging, undifferentiated SH-SY5Y cells exhibited their characteristic epithelial-like phenotype with relatively fewer peripheral projections (Fig. 1b; Day-0#). On differentiation day 2, the cells displayed initiation of neurite growth and a tendency to form small clumps in low-density cultures (Fig. 1b; Day-2#). On differentiation day 4 (two days in low serum with RA), the neurites became prominent, and cell aggregates loosened while multiplication continued (Fig. 1b; Day-4). On differentiation day 6, the cells were more dispersed, their proliferation was stopped, while their neurite extension was continued (Fig. 1b; Day-6). From differentiation day 8 to day 16, neurite extension was further continued, the cell bodies became smaller, and cells aligned after one another forming chain-like neuritic bridges, known as axonal alignments (Fig. 1b; Day-8 to Day-16). On differentiation day 20 (18 days with RA), a well-elongated neuritic system was observed; some cell bodies of differentiated SH-SY5Y cells aggregated and formed islets, while the rest remained as satellites (Fig. 1b; Day-20). The neurites were further elongated and branched. The cell-body islets were further tightened, and the neuritic bridges among them were thickened (Fig. 1b; Day-27). In contrast, the undifferentiated cells that were regularly passaged in 15% serum retained their proliferative competence and morphology throughout the observation period (Fig. 1b; Day-27#).

### Expression of APP throughout the differentiation

**Figure 2:**
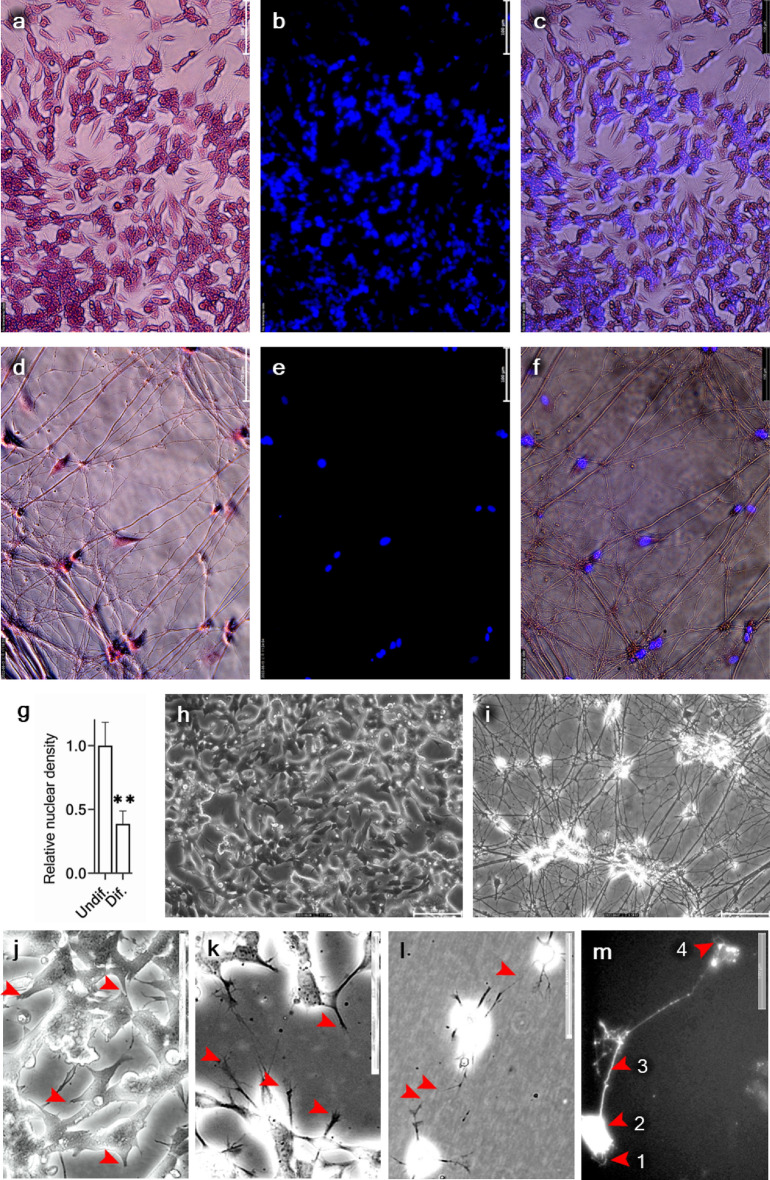
Representative images of the discrete morphology and cell distribution pattern of undifferentiated and differentiated SH-SY5Y cells. SH-SY5Y cells in undifferentiated (**a**–**c**) and differentiated (**d**–**f**) states are shown. The cells are in the undifferentiated form (at day 0), exhibiting densely grown and proliferating morphology (**a**–**c**). The cells are in the differentiated form, exhibiting loosely spread and mature neuron morphology (**d**–**f**). Eosin-stained cytoplasmic and dendritic components (**a** and **d**) and Hoechst-labeled nuclear components (**b** and **e**) were merged (**c** and **f**) to match the soma and nuclei density. The relative nuclear density calculated based on the representative images of undifferentiated and terminally differentiated cells was significantly lower in the terminally differentiated culture (**g**). Live, unstained, phase contrast images of undifferentiated (**h**) and differentiated (**i**) states are also shown. Images used to retrieve the nuclear density are shown in Fig. S11. Detailed peripheral differences among immature, developing, and post-mitotic (mature) SH-SY5Y cell morphological features were also detected. Densely grown, proliferating (undifferentiated) SH-SY5Y cells (**j**). Round or polygonal-shaped cells having retracted phenotype. Yet, they are not polarized and pose relatively fewer and shorter non-dendritic projections (arrowheads). Early differentiating cells with elongated and polarized cell bodies (**k**). Extensions of short neurites from the cell bodies and developing growth cones at the tips of the growing neurites are shown (arrowheads). Early differentiating cells in low-dense culture are extending their growth cones, sensing and making connections (arrowheads) with each other in free space (**l**). An isolated individual post-mitotic neuron (**m**) visualized assisted with its fluorescent labeling. Arrowheads denote, 1: Dendrites, 2: Soma/ Perikaryon, 3: Axon, and 4: Telodendria/ axon terminal. Images were taken using a Leica phase contrast inverted microscope (×20). Scale bars represent 100 μm. The data were considered statistically significant at *p* < 0.05. The significance level was represented as "**": *p* ≤ 0.01. Error bars represent the mean SD (n = 3).

As described above, cell sampling was performed at each time point for mRNA expression analysis. APP mRNA expression was analyzed using reverse transcription-quantitative polymerase chain reaction (RT-qPCR) at each differentiation time point in the differentiation series (Figs. 1c, 2e and S1). The expression was analyzed by targeting three loci in the mRNA/cDNA transcript: the coding region of APP in the extracellular domain region (APP_ED_), the 3′ untranslated region (APP_3ʹUTR_), and the exon 7 and 8 regions (APPv1_770_). Both targeted loci, APP_ED_ (Fig. 1c) and APP_3ʹUTR_ (Fig. 1d), displayed a similar expression pattern throughout the experiment. The lowest APP expression was observed in undifferentiated SH-SY5Y cells. Up to differentiation day 20, an upregulatory trend was observed for both total APP and APPv1_770_. By day 27, the amount of total APP had further risen (Figs. 1c,d, S1a and b), while the expression of APPv1_770_ declined (Figs. 1e and S1c).

### Morphological features of the terminal differentiation state

**Figure 3:**
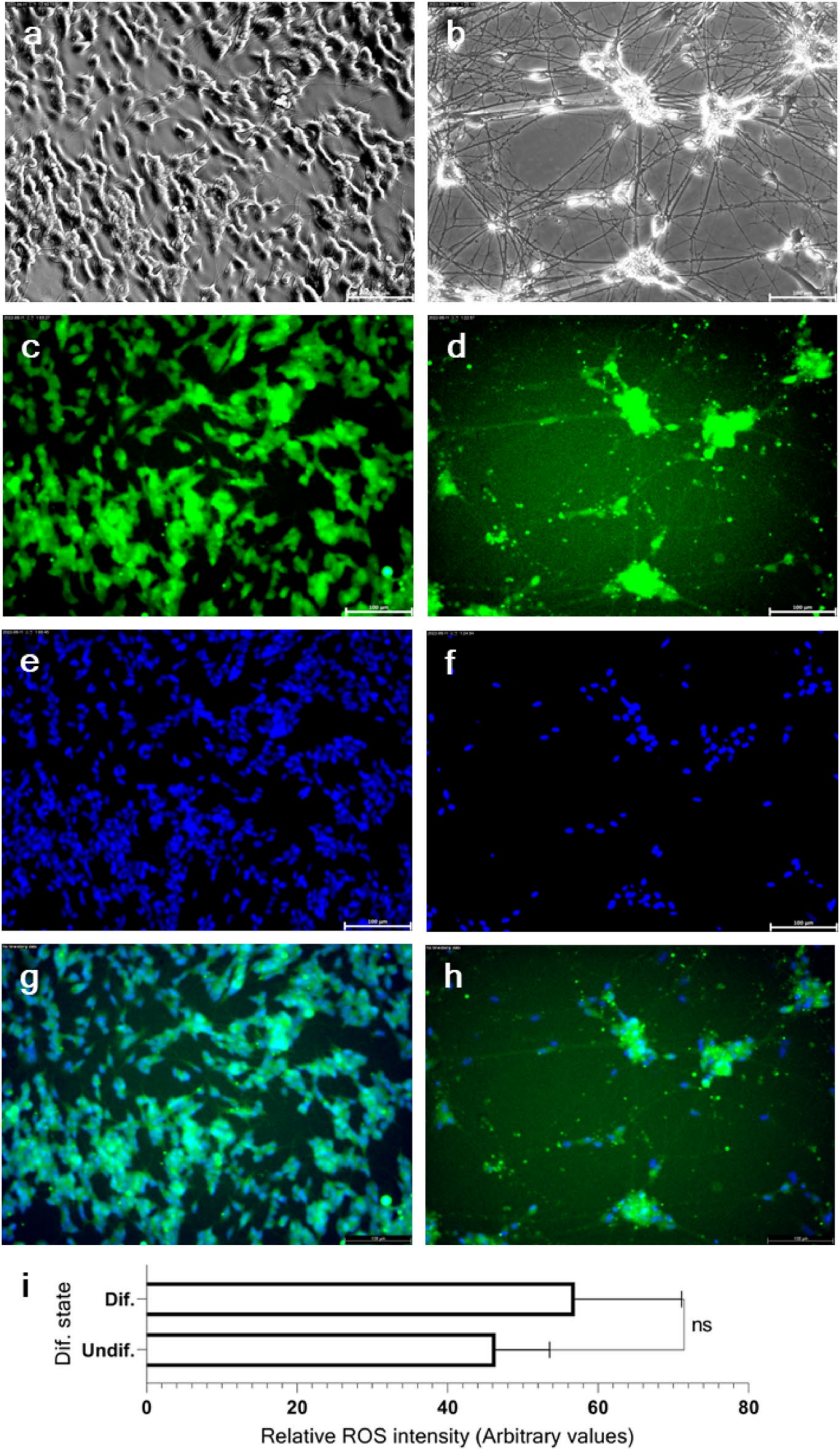
Comparison of ROS activity in undifferentiated and differentiated SH-SY5Y cells. The fluorescence ROS marker H2DCFDA was used to stain immature neuroblastoma cells (**a**,**c**,**e**,**g**) and the terminally differentiated neurons (b, d, f, h). The phase-contrast images (**a** and **b**), the green fluorescence signal from oxidized DCF (**c** and **d**), counterstaining with Hoechst dye for nuclear visualization (**e** and **f**), and merged fluorescence signals (**g** and **h**) were shown. Relative ROS abondance, calculated based on representative images of undifferentiated and terminally differentiated cells, was not significantly different (**g**). The scale bar represents 100 μm. The statistical significance at p < 0.05, represented as "ns": p > 0.05 (not significant). Error bars represent the mean SD (n = 3).

**Figure 4:**
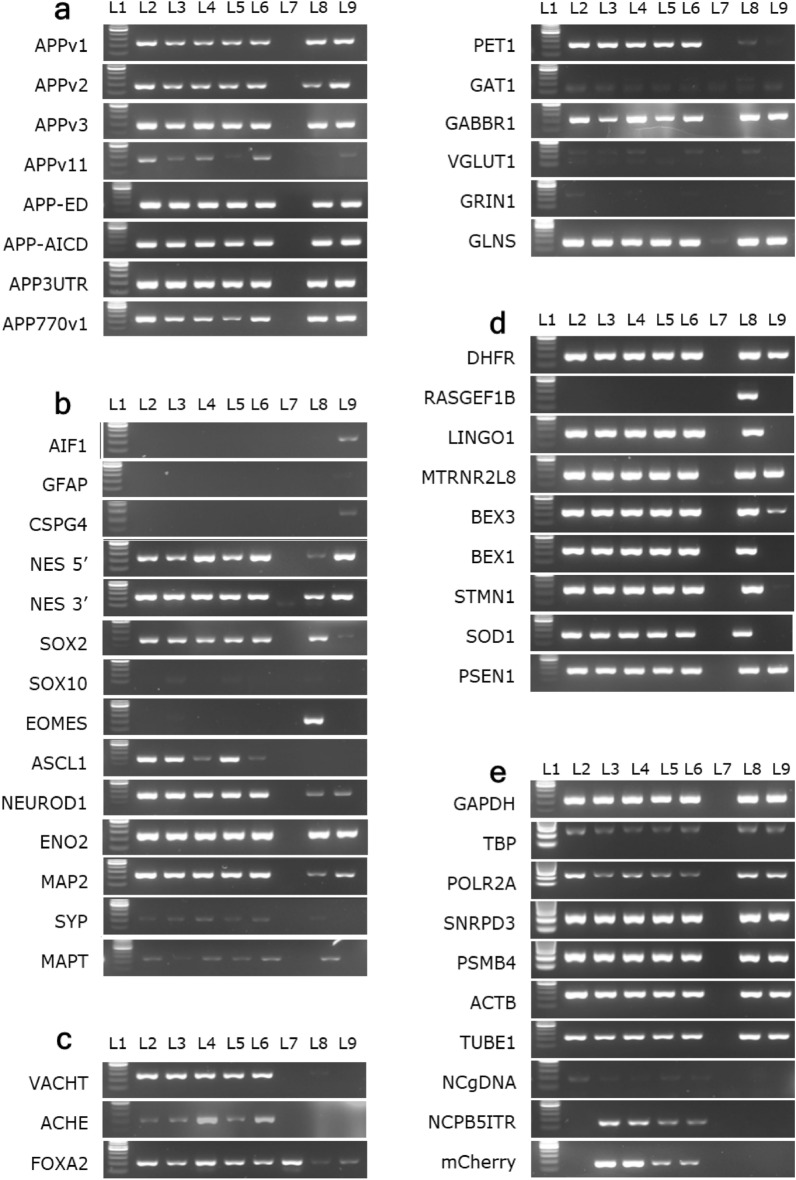
The QPCR targets used. Agarose gel (2%) images of all the QPCR targets used in this experiment. The PCR conditions were identical to the RTQPCR conditions described in the text. In each gel image, 100 bp molecular weight marker is on the Lane 1 (L1). The molecular weight of the bottom most band is 100 bp, and the top bright band is 500 bp. The amplicon sizes of the PCR targets are ~ 200 bp. PCR was performed using the cDNA derived from different types of cells. The corresponding PCR bands are on rest of the lanes as follows: Lane 2 (L2)—undifferentiated and highly proliferative wild-type SH-SY5Y cells. Lane 3 (L3)—undifferentiated Mock/RFP SH-SY5Y cells. Lane 4 (L4)—differentiated Mock/RFP SH-SY5Y cells. Lane 5 (L5)—undifferentiated TgAD/EGFP SH-SY5Y cells. Lane 6 (L6)—differentiated TgAD/EGFP SH-SY5Y cells. Lane 7 (L7)—PCR blank performed without cDNA. Lane 8 (L8)—wild-type 293T cells. Lane 9 (L9)—Heterogenous human endometrium-derived stromal cell cDNA. (**a**) Endogenous and transcript-group-targeted PCR of APP gene (Target info. in Table S3 and original gels are presented in Supplementary Fig. S9). (**b**) Neuron differentiation state marker-targeted PCR (Target info. in Table S4 and original gels are presented in Supplementary Fig. S10). (**c**) Neurotransmitter marker-targeted PCR (see bottom left & top right for complete set) (Target info. in Table S5 and original gels are presented in Supplementary Fig. S11). (**d**) SH-SY5Y cell differentiation state responsive gene-targeted PCR (Target info. in Table S6 and original gels are presented in Supplementary Fig. S12). (**e**) The housekeeping genes, gDNA, and transgenic markers used in PCR and RTQPCR (Target info. in Table S7 and original gels are presented in Supplementary Fig. S20).

In our first observation of the differentiation series, we speculated that a benchmark state for terminal differentiation may lie somewhere between differentiation days 20 and 27. After further optimization, differentiation day 24 was established as the benchmark for terminal differentiation. Subsequently, two time points were selected for sampling: differentiation day 0 to represent the undifferentiated state and differentiation day 24 to represent the differentiated state. A significant variation was observed in the spatial distribution of undifferentiated and differentiated SH-SY5Y cells. The undifferentiated cell population showed a distinct densely grown morphology (Figs. 2a, 3c), whereas the differentiated cell population showed a characteristically dispersed morphology (Figs. 2d, 3f). Thus, resulting a disproportionate soma density between the two cell types (Figs. 2g and S2). These differentiated neurons remained healthy and intact throughout the observation period (32 d) (Figs. 2h and 3i). Furthermore, we observed discrete morphological features among immature, developing, and post-mitotic SH-SY5Y cells (Figs. 2j, 4m). Finally, we compared the reactive oxygen species (ROS) activity of both undifferentiated and differentiated neurons (Fig. 3a–h) and observed similar oxidized 2ʹ,7ʹ-dichlorofluorescein (DCF) intensities in both cell types (Fig. 3i).

### Expression of APP transcript variants

**Figure 5:**
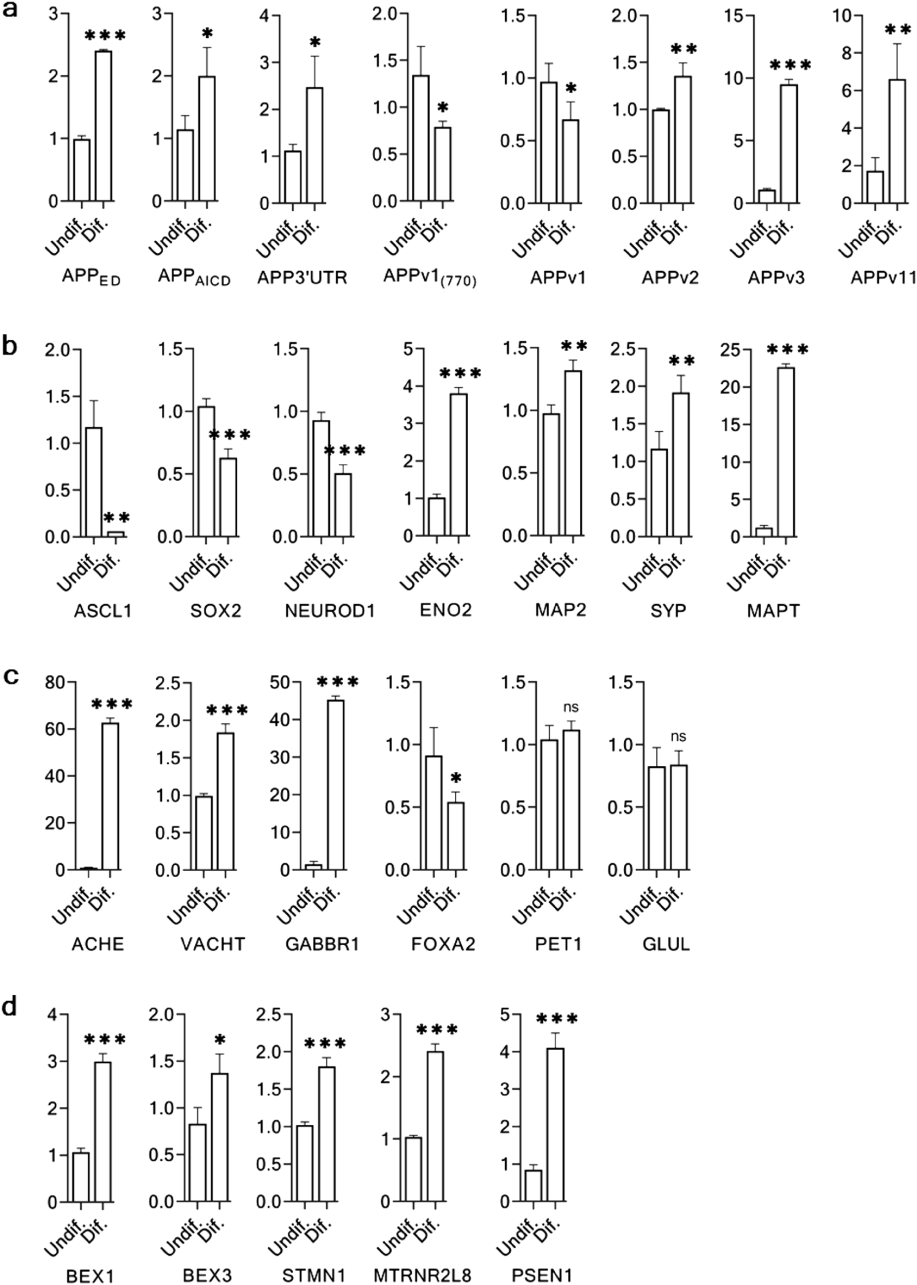
The differentiation state-dependent marker expression in SH-SY5Y cells. Densely grown, proliferating, undifferentiated phenotype (day-0 according to the differentiation procedure) denoted as Undif. and 22-day differentiated phenotype (day-24 according to the differentiation procedure) denoted as Dif. were analyzed for the expression of their differentiation state-dependent markers. Each vertical axis represents the corresponding relative gene expression fold value (arbitrary). (**a**) Transcript variant diversity of APP mRNA in differentiated SH-SY5Y cells. In the differentiated SH-SY5Y cells, the expression of total APP by targeting its common extracellular domain (APPED), intracellular domain (APPAICD) and 3ʹUTR (APP3ʹUTR) showed a significant up-regulation. The APP transcript variant 1 showed a significant down-regulation (APPv1(770) and APPv1), while the APP transcript variant 2 (APPv2), the APP transcript variant 3 (APPv3) and the APP transcript variant 11 (APPv11) showed a significant up-regulation. (**b**) Neuronal progenitor and mature marker expression in differentiated SH-SY5Y cells. The immature neuron markers, ASCL1, SOX2 and NEUROD1 were downregulated and mature neuron markers ENO2, MAP2, SYP, and MAPT were upregulated in differentiated neurons. (**c**) Neurotransmitter marker expression in differentiated SH-SY5Y cells. Common cholinergic neuronal markers, ACHE and VACHT, along with a GABAergic marker, GABBR1 were upregulated compared to other non-cholinergic markers, FOXA2, PET1 and GLUL in differentiated neurons. (**d**) AD-responsive marker expression in differentiated SH-SY5Y cells. The expression of neuron-expressing, AD and brain stress triggered genes BEX1, BEX3, STMN1, MTRNR2L8, and PSEN1 in differentiated neurons. The data were considered statistically significant at *p* < 0.05. The significance levels were represented as "ns": *p* > 0.05 (not significant), "*": *p* ≤ 0.05, "**": *p* ≤ 0.01, and "***": *p* ≤ 0.001. Error bars represent the mean SD (n = 3).

To analyze the expression of the APP transcript variants, we categorized the APP transcript variants into four groups based on the presence or absence of exons 7 and 8 (Tables 2, S1, and S2). Each group contained one of the three major APP transcript variants (transcript variants 1, 2, or 3). A minor variant, transcript variant 11, was classified as group 4. All groups were targeted using transcript variant-specific primers (Fig. 4a and Table S3). The total APP mRNA expression was analyzed by targeting the transcript in three locations: the extracellular domain (Fig. 5a APP_ED_), intracellular domain (Fig. 5a APP_AICD_), and 3′UTR (Fig. 5a APP_3′UTR_). In differentiated cells, the total APP mRNA expression nearly doubled for all three expression targets (Fig. 5a). Alternatively, exons 7 and 8-targeted primers indicated a significant reduction of expression (Fig. 5a APPv1_770_ and Fig. 5a APPv1). A similar expression pattern was observed in TgAD SH-SY5Y cells, except in Group 1, which was upregulated along with other gene targets (Fig. S3). After differentiation, the expression of all transcript-variant groups was upregulated (Fig. 5a APPv2, Fig. 5a APPv3, and Fig. 5a APPv11) except for Group 1, which was downregulated (Fig. 5a APPv1). Specifically, the expression in Groups 3 and 4 increased 8- and 4-fold, respectively (Fig. 5a APPv3 and Fig. 5a APPv11).

**Table 2:** Major groups of APP transcript variants categorized by diversity in exon 7 and 8.

Groups	Group-1	Group-2	Group-3	Group-4
Presence of exon 7 and/or exon 8	Both 7 and 8	7 only	–	8 only
Presence of KPI and/or Ox-2 domains	KPI, Ox-2	KPI	-	Ox-2
Transcript variants	1, 6, 8	2, 4, 9	3, 5, 7, 10	11
Major transcript variant	1	2	3	–

Details of the APP mRNA transcript variants and protein isoforms are mentioned in Tables S1 and S2.

### Neuron maturation and neurotransmitter marker expression in differentiated SH-SY5Y cells

Immature and mature neuronal markers were screened using primers targeting the differentiation states (Fig. 4b and Table S4) in differentiated cells. Compared with those in the undifferentiated state, the tested immature neuron markers ASCL1, SOX2, and NEUROD1 were downregulated and mature neuron markers ENO2, MAP2, SYP, and MAPT were upregulated in differentiated neurons (Fig. 5b). Furthermore, we analyzed the expression of neurotransmitter markers in the differentiated neurons (Fig. 4c and Table S5). The cholinergic neuronal markers ACHE and VACHT were highly upregulated along with the GABAergic marker GABBR1, whereas other non-cholinergic markers were downregulated (FOXA2) or remained neutral (PET1 and GLUL) (Fig. 5c). In line with wild-type cells, a similar pattern of neuronal maturation and neurotransmitter marker expression was observed in the TgAD SH-SY5Y cell line (Figs. S4 and S5).

### AD responsive genes

Furthermore, we analyzed several neurons expressing AD and brain stress-related genes, BEX1, BEX3, STMN1, MTRNR2L8, and PSEN1 (Fig. 4d and Table S6). All the genes were upregulated in the differentiated state (Fig. 5d). A similar expression pattern was also observed in the differentiated TgAD SH-SY5Y cells (Fig. S6).

## Discussion

This study presents an optimized RA-mediated neuronal differentiation protocol for the SH-SY5Y cell line and demonstrates the behavior of the AD central gene, APP, during the transition from the undifferentiated to the terminal differentiated state. More importantly, the study offers a framework for the differentiation process of the SH-SY5Y cell line and proposes a differentiated state-sensitive marker system based on the APP transcript variant dynamics aiming to improve the sensitivity and accuracy of studies employing classical neuronal differentiation markers.

The SH-SY5Y cells produce a nearly homogeneous neuron-like cell population upon RA/BDNF-induced differentiation with higher efficiency^25^. In contrast, other competitive human neuronal cell lines, such as NT-2, require complex differentiation procedures and yield a heterogeneous population of neural lineage cells^26^. Neurons exist in a range of differentiation states, and neuronal progenitors pass through a series of stages during the differentiation process. In neurons, the identification of the exact state of differentiation allied with its morphology is crucial because morphology mostly reflects the physiology, which shapes their functional and structural properties. However, there is no properly established morphological boundary or benchmark for the terminal differentiation of SH-SY5Y cells. The time required to reach the final differentiation state varies greatly among the different procedures describing the differentiation of SH-SY5Y cells. We closely monitored neuronal morphological alterations on consecutive days and photographed and reserved samples for further analysis. Therefore, the differentiation stages described in this experiment can be compared with those described in other studies. Additionally, the oxidative activity of cells is an alternative indicator for their viability and metabolism^27^. Similar levels of oxidative stress were observed at the end of the experiment in both undifferentiated and differentiated cells; this suggests that they had similar levels of metabolic activity throughout the study, despite exposure to long-term culture and differentiation conditions. This finding is significant as it enables researchers to define the differentiation state more precisely, which in fact improves the accuracy and reliability of future experiments.

Multiple studies describing the differentiation of SH-SY5Y cells have demonstrated morphologically inconsistent final differentiation states and reported considerably varying definitions of their boundaries of differentiation (Table 1), In these studies the time taken to appear terminal morphology was varied from day 1^3^ to day 40^17^. Most studies have relied on the expression of certain neuronal markers to indicate differentiation^7,10^. However, identifying a broadly accepted standard for determining the exact state of differentiation is difficult. A standardized differentiation state for SH-SY5Y cells would considerably improve the reproducibility and reliability of the experiments. In the present study, we carefully defined the "true maturity" state of SH-SY5Y cells, in conjunction with the corresponding terminal morphological and differentiation states.

To develop a terminal differentiation state-sensitive marker system, we used the gene, APP. In vitro and in vivo expression of APP gradually increases during neuronal maturation^28,29^. To determine the total APP gene expression in each differentiation state, we targeted both the coding region and 3ʹUTR separately using three primer sets: APPED, APP3ʹUTR, and APP770v1 (Table S3). Expression values of both APPED and APP3ʹUTR represent the total APP mRNA expression folds in the cells while APP770 represents only the transcript variant group-1 which includes the major variant, the transcript variant-1 (Table 2). During differentiation, the expression patterns of APPED and APP3ʹUTR closely followed each other and resembled earlier findings, showing that the APP level gradually rose toward maturity. Another feature of APP is isoform diversity among different types of cells. Among the main isoforms, APP695 is highly expressed in the hippocampus, cerebral cortex, and amygdala^23^. In contrast, APP770 is expressed systemically and declines with neuronal or brain maturity^22^. In our initial assessment, we compared the expression levels of total APP mRNA and APP transcript variant 1. Predictably, we observed a steady decline in the expression of transcript variant 1 relative to total APP from differentiation day 16. Conversely, the total APP expression, largely contributed by APP transcript variant 3 has increased.

APP transcript variant diversity is largely confined to its exons 7 and 8 which contain a Kunitz protease inhibitor (KPI) domain^30,31^ and an orexin 2 (OX2) receptor extracellular domain (Ox-2). The Kunitz protease domain can inhibit a wide range of serine proteases^32^, while Ox-2 plays a role in ligand binding and receptor activation^33^. The functional, structural, and spatiotemporal diversity among the three main isoforms, APP700, APP751, and APP695, is mainly attributed to the presence or absence of their KPI domain^34–36^. Processing of APP yields complex protein processing outcomes, complicating the accurate comparison of protein expression levels. In the present study, we relied on qPCR, which measures gene expression accurately and independently from the protein processing barriers. Parallel to morphological observations, we studied APP gene expression at each stage of differentiation.

To determine the transcript variant expression dynamics, we categorized the APP transcript variants into four groups based on the status (presence or absence) of the KPI and the subsequent Ox-2 domains that reside in exons 7 and 8 (Table 2). In this grouping, the major transcript variants (transcript variants 1, 2, and 3) were confined to groups 1, 2, and 3. Group 4 had a single entity, transcript variant 11. We used four sets of transcript variant-specific exon junction-targeted primers (Table S3). These primers were used for PCR and qPCR to amplify each transcript variant group. In line with previous reports^37^, we observed an increase in total APP expression during the differentiation series and in the 24-day differentiated SH-SY5Y cells.

**Figure 6:**
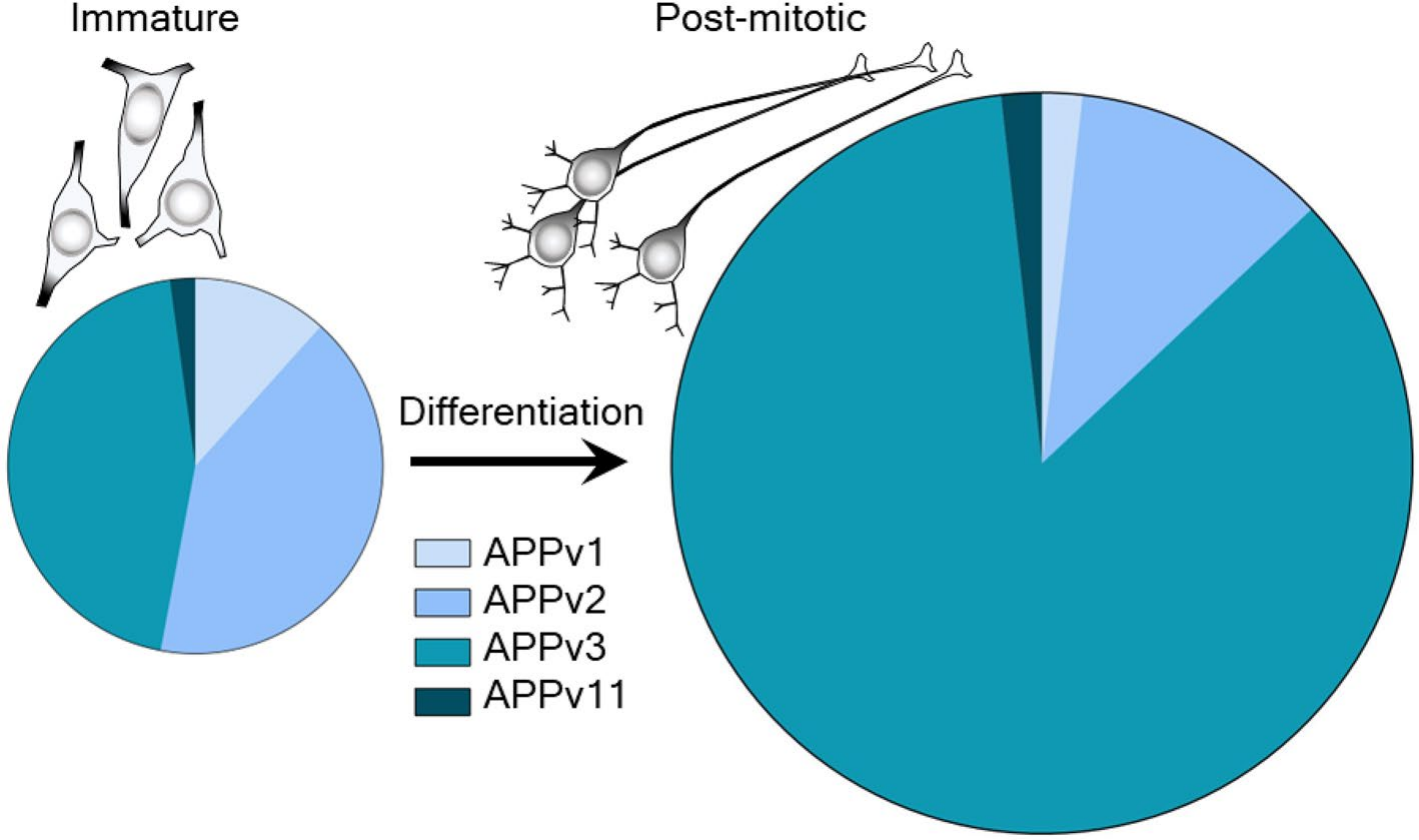
Graphical representation of differentiation state associated, quantifiable (approximate values) transcript-variant dynamics (Among APPv1, APPv2, APPv3, and APPv11) of APP. Relative transcript variant abundance of the immature (proliferative) SH-SY5Y cells in undifferentiated state (Left) and the post-mitotic neuron-like SH-SY5Y cells at *in-natura* terminal differentiated state (Right). The pie charts reflect the relative expression levels of each gene variant as a share of the total expression for both states, highlighting the variance in transcript distribution between the undifferentiated and differentiated states. Note the disproportionation of APPv1 (fallen) and APPv3 (risen) from left to right (for a detailed overview, see Fig. S14).

APP transcript variant 1 (APP770) is the dominant form of APP in non-neuronal cells and neural progenitors, and its expression is often downregulated in mature neurons^22,37^. We noted a declining trend in APP770 expression during the later phases of the differentiation series and in 24-day differentiated SH-SY5Y cells; this proves that the 24-day differentiated cells were well-differentiated into their mature states. Conversely, APP695, the main isoform that lacks the KPI domain and is found mostly in the brain and mature nerve cells, is upregulated during neuronal maturation^23,24^. It could also serve as an effective marker candidate for neuronal maturation. We observed a steady increase in APP695 expression during differentiation. Accordingly, the expression of the APP695 was high in the 24-day differentiated SH-SY5Y cells; this confirms that the 24-day differentiated cells were well-differentiated into their mature state. APP transcript variant 11 (APP714) was also highly upregulated in the differentiated state. Apart from the group 3 entities, APP transcript variant 11 is the only APP transcript variant that does not bear the KPI domain. Based on these outcomes, we propose the expression dynamics of APP as a precise indicator of neuronal maturity. Consequently, we have developed a novel APP splice variant-dependent marker system, guided by the differential expression patterns of APP transcript variants 1 and 3. Specifically, as depicted in Fig. 6, APP transcript variant 1 exhibits downregulation, whereas transcript variant 3 shows upregulation in the mature neurons derived from differentiated SH-SY5Y cells.

We confirmed the differentiation of SH-SY5Y cells by analyzing the gene expression profiles of the differentiated neuron-specific marker genes ENO2, MAP2, SYP, and MAPT. All differentiation markers were significantly upregulated in differentiated SH-SY5Y cells compared to those in undifferentiated cells. ENO2, also known as neuro-specific enolase (NSE), is involved in neuronal glycolysis and is highly expressed in mature neurons^22^. The neuron cytoskeletal structural protein MAP2, mainly expressed in dendrites, is used as a common marker for dendritic development and thus represents neuronal maturity^22^. SYP is a synaptic vesicle glycoprotein mainly found in presynaptic terminals of the neuroendocrine system and is highly expressed in mature neurons^22^. MAPT is a microtubule-associated structural protein abundant in the axons of mature neurons. It is involved in axonal transport, and its dysfunction is associated with several neurodegenerative diseases, including AD^22^. The upregulation of the above-mentioned mature neuron marker genes in differentiated SH-SY5Y cells indicated that these cells gained mature neuronal properties within our differentiation timeframe.

We then analyzed several genes involved in the maintenance of pluripotency and early neurogenesis. Undifferentiated cells were characterized by the expression of SOX2, ASCL1, and NEUROD1 genes. These immature neuronal marker genes were downregulated in the differentiated state, indicating a shift from a proliferative stem-like state to a mature phenotype. SOX2 is an essential transcription factor involved in maintaining the pluripotency of undifferentiated stem-like cells, including embryonic and induced pluripotent stem cells^38^. SOX2 is expressed in adult neural stem cells and other lower hierarchy neural progenitors and downregulated in post-mitotic neurons^39^. ASCL1 is a marker for the transient differentiation of neural progenitor cells, and its expression is downregulated in differentiated neurons^40^. NEUROD1 is an early embryonic-expressed gene involved in neural differentiation^41^. As maturity progresses, the expression of NEUROD1 is confined to specific regions and certain types of neurons, such as the pyramidal cells in the brain^42^, thus downregulating its expression in most types of neurons, including in the SH-SY5Y cells^16^.

In addition to that, we also focused on the expression of neurotransmitter markers during SH-SY5Y cell differentiation. Our results showed that the cholinergic neuronal marker genes, ACHE and VACHT, were highly upregulated in differentiated cells. Multiple studies have reported that RA-induced SH-SY5Y cell differentiation directs dopaminergic phenotypes^2,3^. Conversely, we observed highly upregulated cholinergic markers in the differentiated cells, consistent with the recent reports of cholinergic differentiation of SH-SY5Y cells^11^. The upregulation of cholinergic markers suggested that differentiated SH-SY5Y cells acquired cholinergic neuronal phenotypes. Notably, a non-cholinergic marker, GABBR1, was also highly upregulated during differentiation; this can be explained by evidence showing GABAB receptor expression in certain cholinergic neurons, particularly those in the habenula region^43^. We observed a significant downregulation of FOXA2, a dopaminergic neuron maturation marker^44,45^, suggesting that the differentiated SH-SY5Y cells may not exhibit dopaminergic phenotypes. This confirms our hypothesis on the cholinergic phenotype of mature SH-SY5Y cells. Finally, we found that PET1 and GLUL expression remained unchanged during differentiation. The expression of PET1 is associated with serotonergic neurons^46^ while that of GLUL is associated with glutamatergic neurons and glial cells, mainly astrocytes^47^. Thus, the differentiated SH-SY5Y cells did not exhibit serotonergic, glutamatergic, or glial phenotypes. The technique we used to differentiate SH-SY5Y cells has successfully directed the cells toward the cholinergic neuron phenotype. Besides, cholinergic neurodegeneration has been widely implicated in AD^48^ and is one of the earliest pathological events^49^, making these differentiated SH-SY5Y cells a probable candidate model for AD studies.

Transcriptomic analysis of AD brains has revealed differentially expressed genes triggered by AD pathology in neurons and in proliferative and mature SH-SY5Y-derived neurons^50,51^.

These genes are associated with mature neurons and respond to Alzheimer's disease, either by upregulation or downregulation and the expression of these genes was previously compared solely between diseased and healthy states in mature neurons^50,51^. To demonstrate how mature neuron-associated AD-responsive genes are involved in neuronal differentiation, we analyzed several genes, including BEX1, BEX3, STMN1, MTRNR2L8, and PSEN1. All mature neuron-associated AD-responsive genes were highly upregulated in differentiated SH-SY5Y cells compared to those in undifferentiated cells. This suggests that mature neuron-associated AD-responsive genes also could be used as alternative markers for neuronal differentiation.

To further validate this novel marker system, we employed a previously adopted 3xAD overexpression system^52^. The 3xAD overexpression system is widely used to overexpress AD-related genes, including in vivo^53^. We created and incorporated the 3xAD system to produce the TgAD-SHSY5Y cell line to assess our marker system. Consistent gene expression patterns were observed in both wild-type SH-SY5Y cells and TgAD-SHSY5Y cells. This additional validation underscores the robustness and applicability of our marker system across different experimental setups.

While undifferentiated SH-SY5Y cells served as a negative control in our study, adding a positive control that closely represents the fully differentiated state of SH-SY5Y cells, such as primary human adult neurons derived from cell culture or clinical brain samples would strengthen the validity of our outcomes. While the dynamics of APP in neuron differentiation are implicated, the link to its transcript variant/isoform patterning is yet to be elucidated. Transcript variant dynamics during SH-SY5Y differentiation discuss our study may shed light towards scientists to find the key role of APP in neuron maturity. Analyzing transcript variant dynamics and conducting differentiation time series analysis of other classical neuronal markers, similar to this study, would be beneficial for future research.

In summary, we differentiate the SH-SY5Y cell line comparatively extended period while continuously observing their morphology along with total APP and APP transcript variant 1 expression dynamics. In our results, we observed a continuous upregulation of total APP, dominated by APP transcript variant 3 while downregulation of APP transcript variant 1 in differentiated SH-SY5Y cells, starting from day 24 and beyond. In the same maturity state, we observed upregulation of total APP, including it’s all the transcript variants (except the transcript variant 1) along with mature neuron-specific markers, cholinergic-type neurotransmitter markers, and mature neuron-associated AD-responsive genes. These findings concluded that APP transcript variants-based, differentiated state-sensitive marker system could be used to effectively characterize the advanced stages of differentiated SH-SY5Y-derived neuronal cells, demonstrating a "true maturity." To the best of our knowledge, this is among the most comprehensive studies on SH-SY5Y differentiation with respect to the expression of the AD central gene, APP. The study findings provide valuable insights into SH-SY5Y differentiation and present new tools for improving the accuracy of future neuronal differentiation experiments.

## Methods

### Materials

The human neuroblastoma cell line, SH-SY5Y (ATCC^®^ CRL­2266™) was purchased from the ATCC (American Type Culture Collection). The SH-SY5Y-Red and SH-SY5Y-Green-3xAD(wt) cell lines were transgenically produced. All DNA manipulations and vector assemblies were performed using kits and enzymes purchased from New England Biolabs (NEB). Unless otherwise specified, all cell culture and transfection reagents were obtained from Invitrogen. RNA extraction and plasmid Maxiprep kits were purchased from QIAGEN, Germany. Reverse transcriptase, qPCR mastermix, and plasmid miniprep kits were purchased from Bioneer (Daejeon, Korea). Nucleic acid concentrations were measured using a Biospec-nano (Shimadzu, Japan) microvolume spectrophotometer. Electrotransfection was performed using the Neon Electroporation Transfection System (Thermo Fisher Scientific). Stocks of all-trans retinoic acid (ATRA) (Sigma R2625) were prepared in DMSO at concentrations of 50 mM (5000×) and 10 mM (1000×). A 1 M (50×) stock solution of potassium chloride (KCl) (Sigma P5405) was prepared in deionized water and filter-sterilized. A 1 M (500×) stock of N6,2ʹ-O-Dibutyryladenosine 3ʹ,5ʹ-cyclic monophosphate (dibutyryl cAMP or db-cAMP) (Sigma D0627) was prepared in sterilized water. A 50 µg/mL (1000×) stock of human brain-derived neurotrophic factor (BDNF) (GenScript Z03208-5) was prepared in final differentiation media (without RA). Except for KCl, all other stock solutions were stored at − 80 °C in single-thaw aliquots.

### Cell culture

The SH­SY5Y, SH-SY5Y-red, and 3xAD(wt)-SH-SY5Y-green cell lines were maintained in a 1:1 mixture of DMEM and F12 medium (Gibco™ cat. 11320033) supplemented with 15% heat-inactivated fetal bovine serum (FBS) (Gibco, cat. 10082147). Except for transfections, 100 U/mL penicillin–streptomycin (Gibco™ cat. 15140148) was included in all the complete culture media. The ability of SH-SY5Y cells to attach to the PEI-coated surfaces was assessed (Fig. S7), and 0.1 mg/mL PEI (Sigma cat. 408727-100ML) was used for plate coating in the differentiation experiment. Cells were cultured in an incubator at 37 °C and 5% CO_2_. Cells were sub-cultivated following the product guidelines, ensuring that confluency remained below 90%. For cryopreservation, the complete medium was supplemented with 5% (v/v) DMSO.

### Expression constructs

The piggyBac (PB) transposon with the pPBmin vector backbone (Fig. S8A) was used to create both integrated expression vectors. Briefly, overlapping DNA sequences of the piggyBac left arm (5′ inverted terminal repeat (5′ITR) and downstream cis element), human cytomegalovirus (CMV) immediate early enhancer and promoter (508 bp), coding sequence of mammalian codon-optimized mCherry gene (708 bp), ribosomal skipping GSG-P2A self-cleaving peptide sequence (66 bp), the hygromycin B phosphotransferase gene (1023 bp), rabbit β-globin polyadenylation signal sequence (56 bp), and piggyBac right arm (3′ inverted terminal repeat (3′ITR) and upstream cis element) with minimal backbone for replication in *E. coli* (pUC ori and β-lac. gene) were amplified using Q5^®^ High-Fidelity DNA Polymerase. The sequences were then assembled using NEBuilder HiFi DNA assembly to produce the mammalian expression piggyBac transposon vector, pPBmin·CMVp·mCherry·P2A·HygR·rβGpA (Fig. S8B). This vector served as the control (mock) vector in transfections and subsequently gave rise to the wt-SH-SY5Y-mCherry (red) cell line. The promoter activity and antibiotic resistance of these newly built expression vectors were evaluated using the SH­SY5Y cell line.

Similarly, several sequences, such as the human ENO2 promoter (1268 bp), MAPT transcript variant 3 (1149 bp, 383 aa), APP transcript variant 3 (2085 bp, 695 aa), CMV promoter (508 bp) directed EGFP (717 bp, 239 aa), and SV40 promoter (358 bp) directed NeoR (795 bp, 264 aa), were sequentially assembled as 2A sequence mediated multi-cistronic manner to result 3xAD vector (Fig. S8C). A transiently expressing transposase vector, sPBo-EGFP (helper), was constructed by combining a transposase with an EGFP reporter (Fig. S8D).

### Transfection and stable cell lines

Each sequence-verified expression plasmid vector was retransformed into chemically competent NEBstable or JM109 Escherichia coli. A single colony was cultured in 100–200 mL LB broth. From this culture, a bacterial pellet of approximately 300 mg was obtained by centrifugation (1500×*g*) for 1 min and used for plasmid maxiprep.

For the electro-transfection mix, 4 μg of the transposon vector was combined with 2 μg of transposase helper in 60 μL volume and then added to 10^6^ cells. Electroporation was performed at 900 V for 30 ms as a single pulse. Antibiotic screening and clone isolation were performed on each TgAD cell line with 100 μg/mL hygromycin B for 10 days (for mCherry/Red mock cells) or 200 μg/mL Geneticin (G418) for 21 days (for EGFP/green 3xAD(wt) cells). The mCherry/Red mock cell line was designated as SH-SY5Y-red, and the EGFP/green 3xAD(wt) cell line as 3xAD(wt)-SH-SY5Y-green.

### Differentiation of SH-SY5Y and SH-SY5Y based transgenic cell lines

The SH­SY5Y cell line was cultured in DMEM/F12 media containing 15% serum. Serum concentration was reduced to 10% of the previous passage prior to cell seeding. The experiment was performed in 0.1 mg/mL polyethyleneimine-coated 12-well plates (4 cm^2^/well). The regular subculture ratio was 1:4. For differentiation, the seeding density was reduced to 1:16 as 12,500 cells cm^−2^ (10^6^ cells/78.5 cm^2^ or (ϕ 100 mm cell culture plate)). Cells were seeded in 600 μL of culture media containing 10% serum at a cell count of 5 × 10^4^/ well (Day-0). Media were replaced every two days until Day 12. After two days (Day 2), the medium serum was reduced to 2.5%. Beginning from Day 2, throughout the experiment, all the media were supplemented with 10 µM RA. On Day 6, medium serum was further reduced to 1%. On Day 12, the serum was totally withdrawn in the final differentiation media and supplemented with 1 × B-27 (Gibco™ cat. 17504044), 20 mM KCl, 50/mL BNDF, and 2 mM db-cAMP. Media refreshing was performed every four days after Day 12, and the cultures were maintained until Day 32. The sampling during the differentiation process is shown in Fig. 1a. Sampling was performed on Day 1 (undifferentiated) and day 24 (differentiated) for the TgAD cells (both SH-SY5Y-red and 3xAD(wt)-SH-SY5Y-green).

### mRNA expression analysis

Each cell sample prepared for RNA extraction was adjusted to a cell count of 0.5–1 × 10^6^, pelleted (1500×*g*, 5 min) in a 1.5 mL microcentrifuge tube, and immediately stored at − 80 °C until the extraction process began. Total RNA was extracted from frozen samples using an RNeasy Mini Kit according to the manufacturer's instructions, and RNA concentrations were measured. First-strand cDNAs were synthesized as 50 μL total reactions. Reaction components were 25 μL of AccuPower^®^ RT 2× mastermix, 2.5 μL (reaction conc. 5 μM) of anchored oligo (18nt, Tm: 37.6–39.3 °C, 100 μM stock) dT16VN (5′ TTTTTTTTTTTTTTTTVN 3′), 1.5 μg of total RNA, and nuclease-free water.

For RT, a 25 μL mixture of total RNA and oligo-dT16VN was incubated at 70 °C for 5 min, then the tubes were transferred into an ice block, and 25 μL of 2× RT mastermix was added. The RT reaction was performed at 42 °C for 1 h, and subsequent RT inactivation was done at 95 °C for 5 min. Finally, the cDNA was diluted 10- to 20-fold in nuclease-free water and stored at − 80 °C until further use.

The RT-qPCR was performed using AccuPower^®^ 2× GreenStar™ qPCR mastermix as 10 μL reactions. Each reaction mixture comprised 2.5 μL of cDNA (1–2 ng/μL), 2.5 μL of primer mix (1 μM of each forward and reverse primer), and 5 μL of 2× mastermix. The PCR cycling conditions were adopted according to the manufacturer's instructions. The efficiency of the RT-qPCR primers was checked by RT-qPCR using serially diluted cDNA and following the equation E = (10(− 1/ − slope) – 1) × 100%. The relative expression fold was determined by the 2^−ΔΔCT^ method^54^. For all quantitative expression analysis, several potential candidate genes, including GAPDH, were tested as housekeeping genes (Fig. 4e and Table S7). Moreover, a transcriptionally inactive genomic locus, the 5ʹ PB transposon element, and the transgenic construct were targeted as control reactions in the qPCR (Fig. 4e and Table S7).

### Differential staining and cell imaging

All cell images were acquired using a Leica DMi8 inverted fluorescence microscope equipped with Leica Application Suite X (LAS X). The morphology of each stably expressing cell line was examined in both live and fixed cells. Red and green fluorescence and phase-contrast images were obtained for each cell line and coculture.

For eosin-Y staining, the cultures were fixed in 4% paraformaldehyde for 15 min, flushed with a 0.5% eosin-Y solution, and then rinsed three times with PBS to remove excess stains. The Cytosoles (somata) were visualized as pink under a bright field microscope.

For reactive oxygen species (ROS) detection, Image-iT™ (Invitrogen, I36007) a 5-(and-6)-carboxy-2ʹ,7ʹ-dichlorodihydrofluorescein diacetate (carboxy-H2DCFDA) based kit was used to stain live cells (green fluorescent).

For nuclear-counterstaining, dead or live cells were incubated at 37 °C for 10 min in 0.5 µg/mL Hoechst 33342 (Invitrogen™, H3570 10 mg/mL). The cells were washed three times with PBS to remove excess stains, and a blue, fluorescent signal was detected.

Images were further processed for quantitative analysis, and the mean gray values were quantified using Neuron J, an image analyzer^55^, was used to analyze cell, nuclear, and dendrite densities and fluorescent intensities.

### Statistical analysis

All data are expressed as the mean ± standard error of the mean (SEM). When comparing the two treatment groups, an unpaired, one-tailed Student's t-test was used.

The Sidak multiple comparison test was performed to compare the treatment groups with the corresponding controls, followed by one-way analysis of variance (ANOVA). For comparing multiple treatment groups, ANOVA was performed, and subsequent post-hoc tests were conducted using Tukey's honestly significant difference (HSD) test. A probability value ≤ 0.05 was considered statistically significant.

### Supplementary Information

Supplementary Information.

## Data Availability

The data of this is available from the corresponding author on request.
