# Supplementary material for: A novel APP splice variant-dependent marker system to precisely demarcate maturity in SH-SY5Y cell-derived neurons
Source: Sci Rep. 2024 May 27;14:12113. doi: 10.1038/s41598-024-63005-y (PMC11130256; doi:10.1038/s41598-024-63005-y)
Supplement: Supplementary file 1 — Supplementary Information. [file 41598_2024_63005_MOESM1_ESM.pdf]

SUPPLEMENTARY INFORMATION

A Novel APP Splice Variant-Dependent Marker System to Precisely Demarcate Maturity in SH-SY5Y Cell-Derived Neurons

D Chanuka M Kulatunga[1], Umanthi Ranaraja[1], Eun Young Kim[2], Ryoung Eun Kim[2], Dong Ern Kim[1], Kuk Bin Ji[1], Min Kyu Kim* [1,2]

[1] Laboratory of Animal Reproduction and Physiology, College of Agriculture and Lifesciences, Chungnam National University, Yuseong-gu, Daejeon, 34134, Republic of Korea.

[2] MK Biotech Inc., Daejeon, Republic of Korea.

* Corresponding author: Min-Kyu Kim, DVM, Ph.D.,
Professor, Department of Animal Science and Biotechnology, College of Agriculture and Life Science, Chungnam National University, Yuseong-gu, Daejeon, 34134, Republic of Korea. Tel: +82 42 821 5773; Fax: +8242 42 825 9754 E-mail: kminkyu@cnu.ac.kr

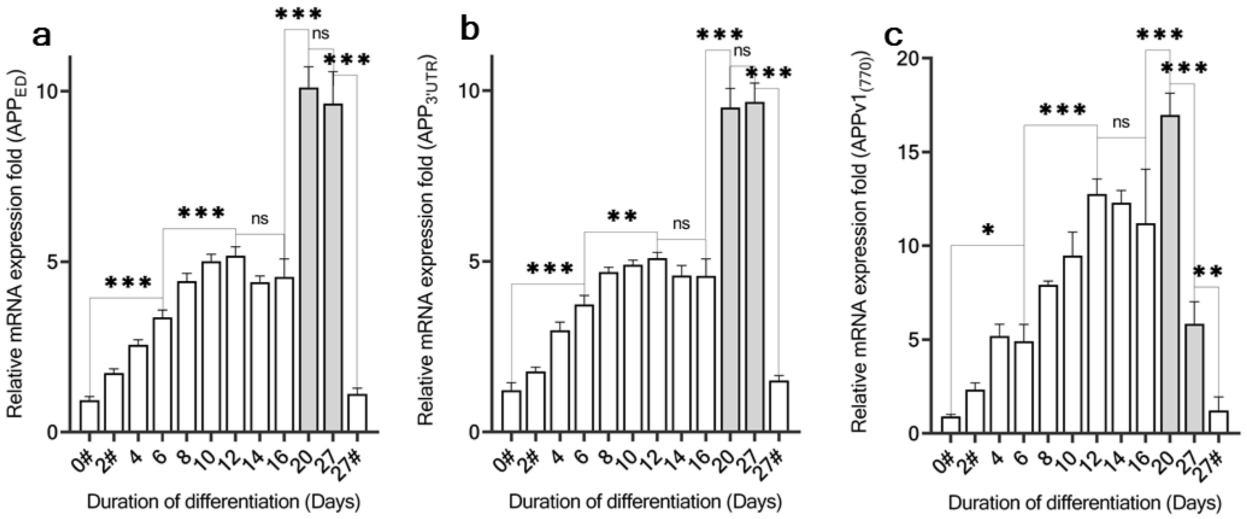

Figure S1: APP mRNA expression dynamics during SHSY5Y differentiation.
The total APP mRNA expression dynamics throughout the differentiation process by targeting the common APP extracellular domain (a) and APP 3'UTR (b) in QPCR. The APP mRNA transcript variant 1 (APP770) expression dynamics throughout the differentiation process by targeting the 7 and 8 exons of APP in QPCR (c). The gene SNRPD3 was used as the reference internal control for the normalization. The cells cultured under proliferative conditions (≥10% serum and without RA supplementation) were marked with "#". The data were considered statistically significant at $p < 0.05$. The significance levels were represented as "ns": $p > 0.05$ (not significant), "*": $p \leq 0.05$, "**": $p \leq 0.01$, and "***": $p \leq 0.001$. Error bars represent the mean SD (n=3).

a

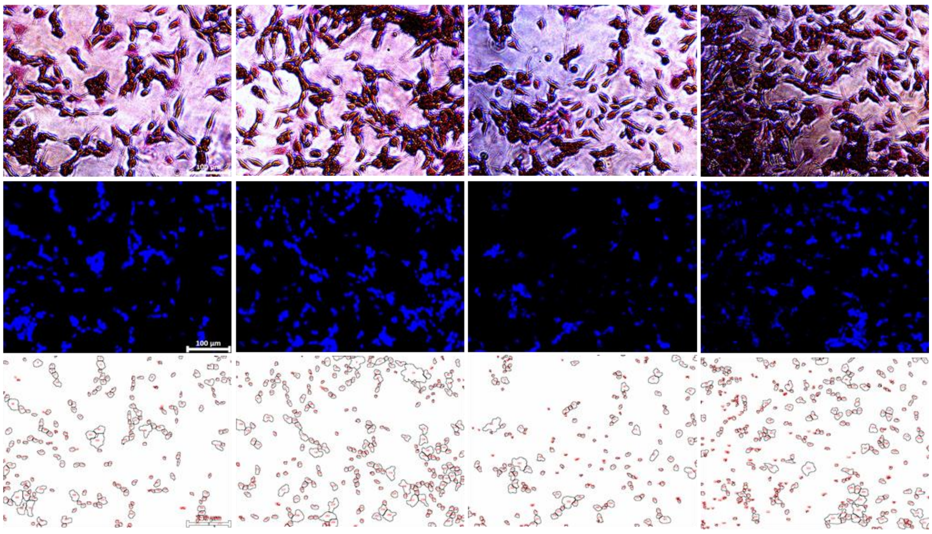

b

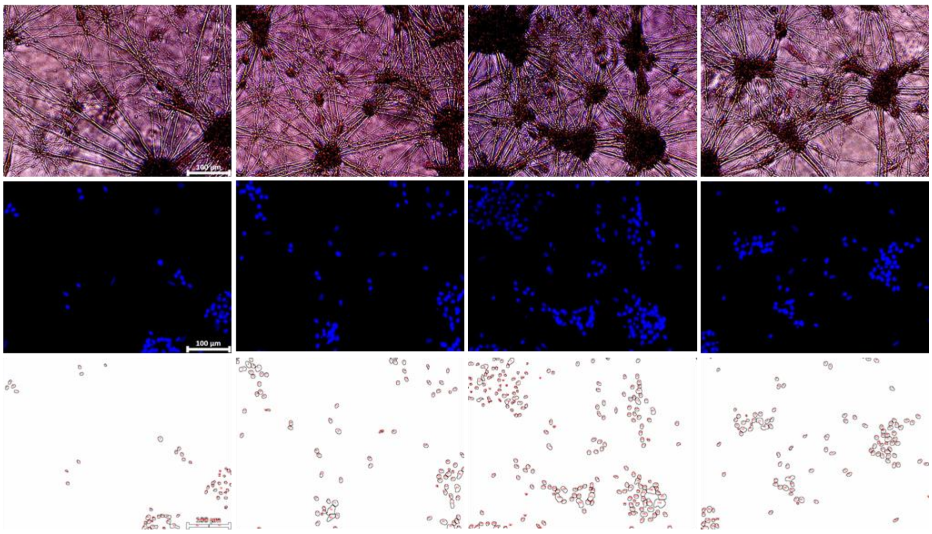

Figure S2: Image-retrieved nuclear/ cell density of undifferentiated and differentiated cells. The data is presented in Figure 3g.

a) Brightfield images of Eosin stained, undifferentiated SH-SY5Y cells (Top), their Hoechst labeled nuclear component (Middle), and its ImageJ generated nuclei distribution map (Bottom).

b) Brightfield images of Eosin stained, differentiated SH-SY5Y cells (Top), their Hoechst labeled nuclear component (Middle), and its ImageJ-generated nuclei distribution map (Bottom). The left to right four sets of images are replicates. The images were taken using Leica phase contrast, inverted, florescent microscope (20x, scale bar; 100 μm).

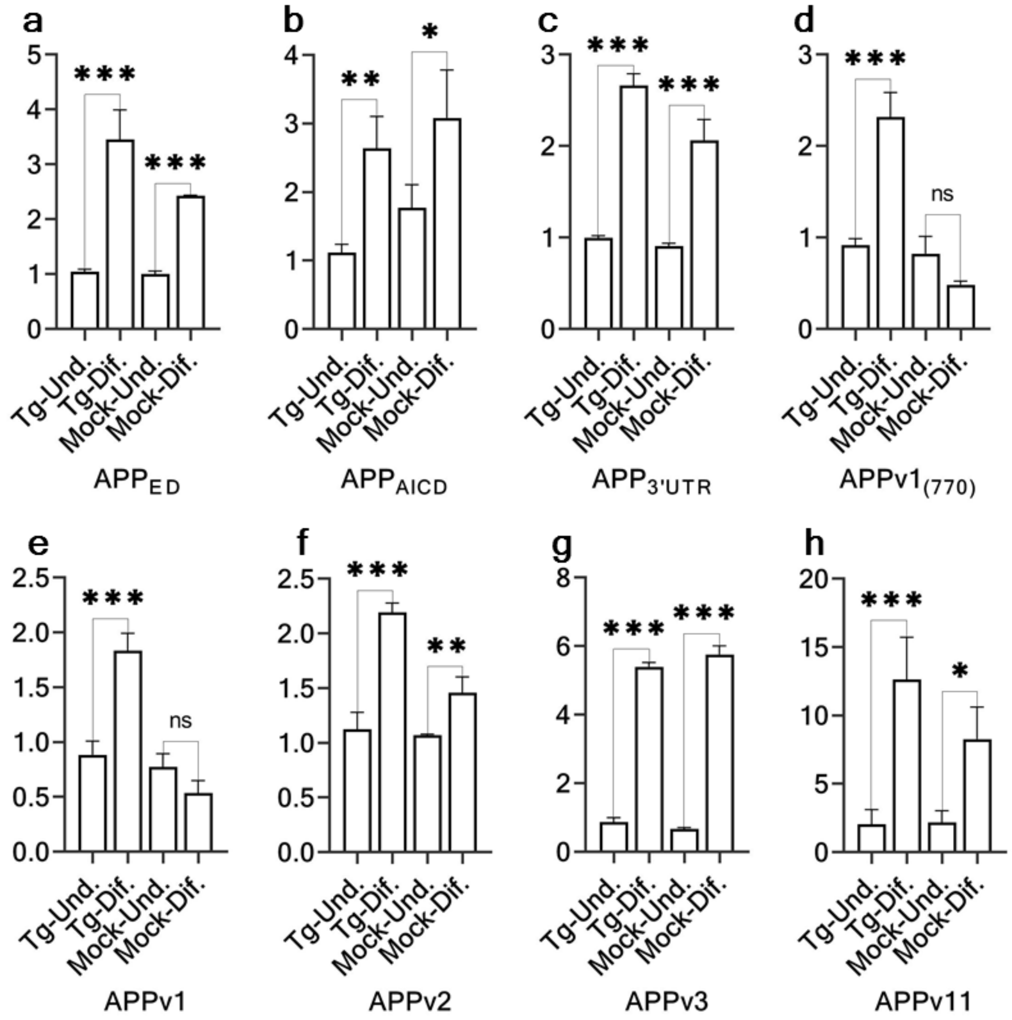

Figure S3: Transcript variant diversity of APP mRNA in differentiated TgAD SH-SY5Y cells. Transcript variant expression of APP in differentiated Tg cells denoted as Tg-Dif. Compared to the densely grown, proliferating, undifferentiated phenotype denoted as Tg-Und.
Corresponding undifferentiated (Mock-Und.) and differentiated (Mock-Dif.) expression data of the Wt/ Mock cell line was presented for comparison. The expression of total APP by targeting its common extracellular domain (APPED), intracellular domain (APPAICD), and 3'UTR (APP3'UTR) showed a significant up-regulation. The APP transcript variant 1 also showed a significant up-regulation (APPv1 (770) and APPv1) along with the APP transcript variant 2 (APPv2), the APP transcript variant 3 (APPv3), and the APP transcript variant 11 (APPv11). The data were considered statistically significant at $p < 0.05$. The significance levels were represented as "ns": $p > 0.05$ (not significant), "*": $p \leq 0.05$, "**": $p \leq 0.01$, and "***": $p \leq 0.001$. Error bars represent the mean SD (n=3).

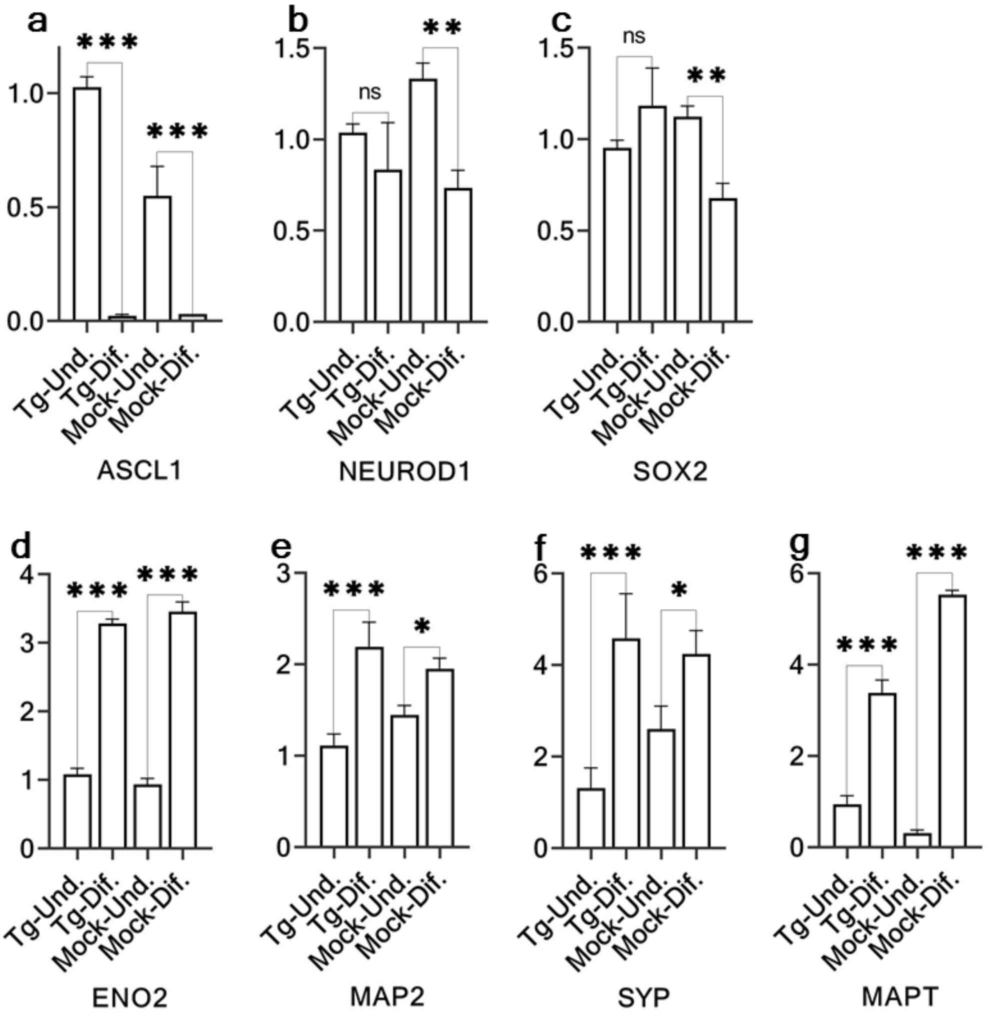

Figure S4: Neuronal progenitor and mature marker expression in differentiated Tg SH-SY5Y cells.

Neuronal progenitor and mature marker expression in differentiated Tg cells denoted as Tg-Dif. compared to the densely grown, proliferating, undifferentiated phenotype denoted as Tg-Und.

Corresponding undifferentiated (Mock-Und.) and differentiated (Mock-Dif.) expression data of the Wt/ Mock cell line was presented for comparison.

The immature neuron markers ASCL1, SOX2, and NEUROD1 were downregulated, and mature neuron markers ENO2, MAP2, SYP, and MAPT were upregulated in differentiated neurons.

The data were considered statistically significant at $p < 0.05$. The significance levels were represented as "ns": $p > 0.05$ (not significant), "*": $p \leq 0.05$, "**": $p \leq 0.01$, and "***": $p \leq 0.001$. Error bars represent the mean SD (n=3).

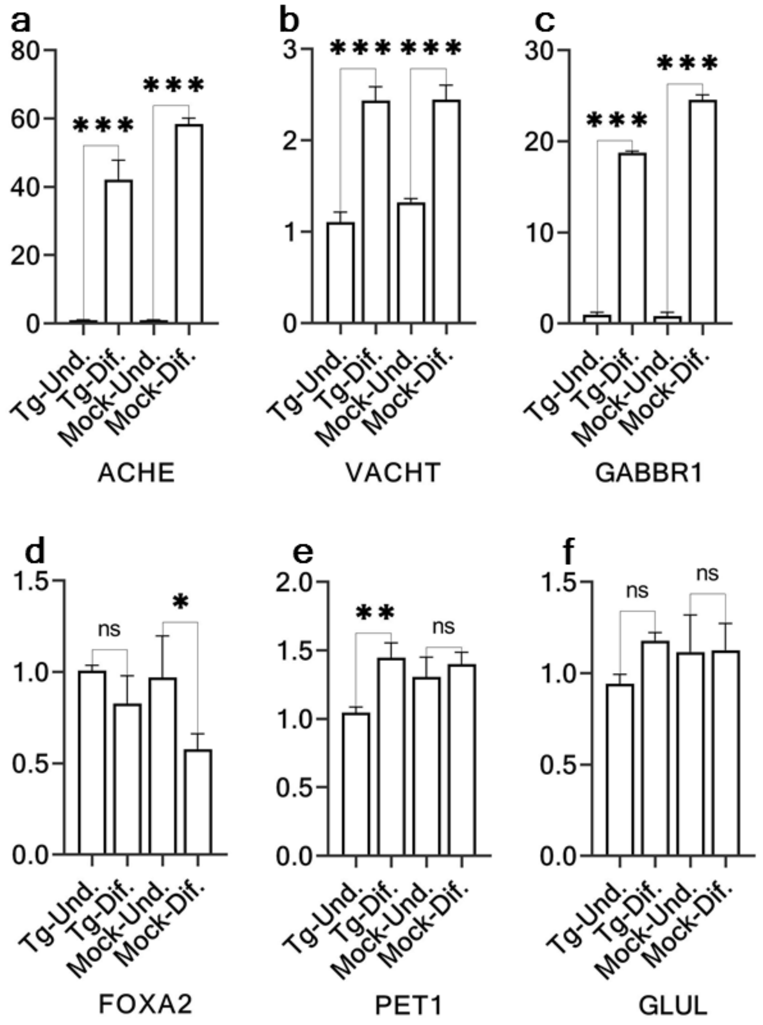

Figure S5: Neurotransmitter marker expression in differentiated Tg SH-SY5Y cells. Neurotransmitter marker expression in differentiated Tg cells denoted as Tg-Dif. compared to the densely grown, proliferating, undifferentiated phenotype denoted as Tg-Und. Corresponding undifferentiated (Mock-Und.) and differentiated (Mock-Dif.) expression data of the Wt/ Mock cell line was presented for comparison. Common cholinergic neuronal markers, ACHE and VACHT, along with a GABAergic marker, GABBR1, were upregulated compared to other non-cholinergic markers, FOXA2, PET1, and GLUL in differentiated neurons. The data were considered statistically significant at $p < 0.05$. The significance levels were represented as "ns": $p > 0.05$ (not significant), "*": $p \leq 0.05$, "**": $p \leq 0.01$, and "***": $p \leq 0.001$. Error bars represent the mean SD (n=3).

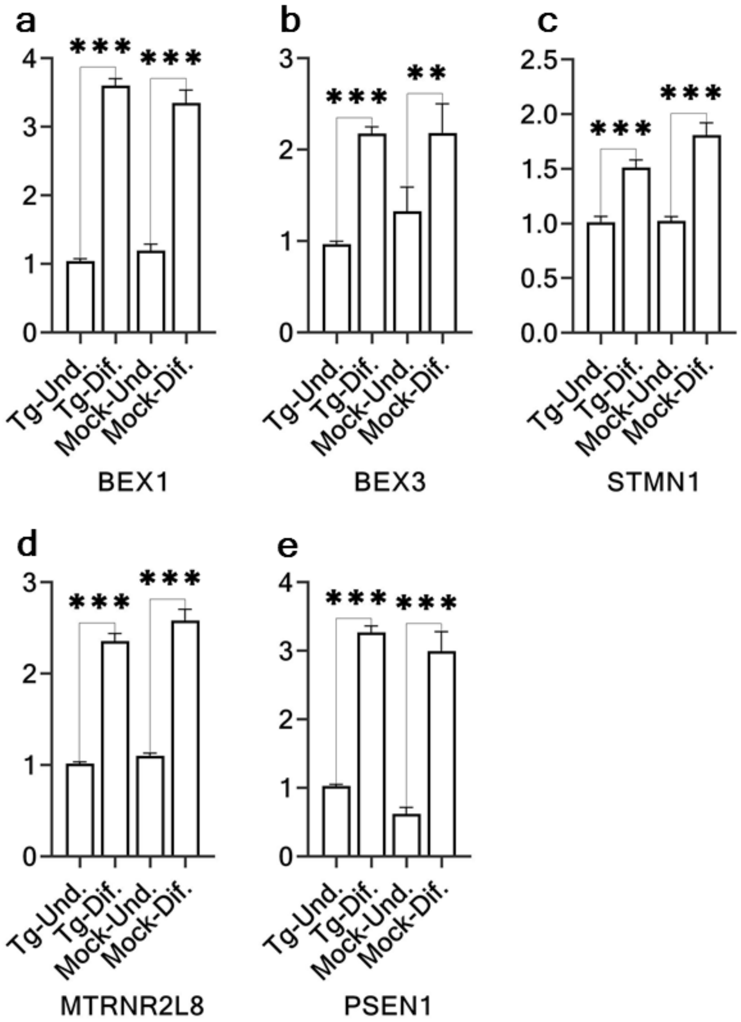

Figure S6: AD responsive marker expression in differentiated Tg SH-SY5Y cells.
AD responsive gene expression in differentiated Tg cells denoted as Tg-Dif. compared to the densely grown, proliferating, undifferentiated phenotype denoted as Tg-Und.
Corresponding undifferentiated (Mock-Und.) and differentiated (Mock-Dif.) expression data of the Wt/ Mock cell line was presented for comparison.
The expression of AD and brain stress triggered genes BEX1, BEX3, STMN1, MTRNR2L8, and PSEN1 in differentiated neurons.
The data were considered statistically significant at $p < 0.05$. The significance levels were represented as "ns": $p > 0.05$ (not significant), "*": $p \leq 0.05$, "**": $p \leq 0.01$, and "***": $p \leq 0.001$. Error bars represent the mean SD (n=3).

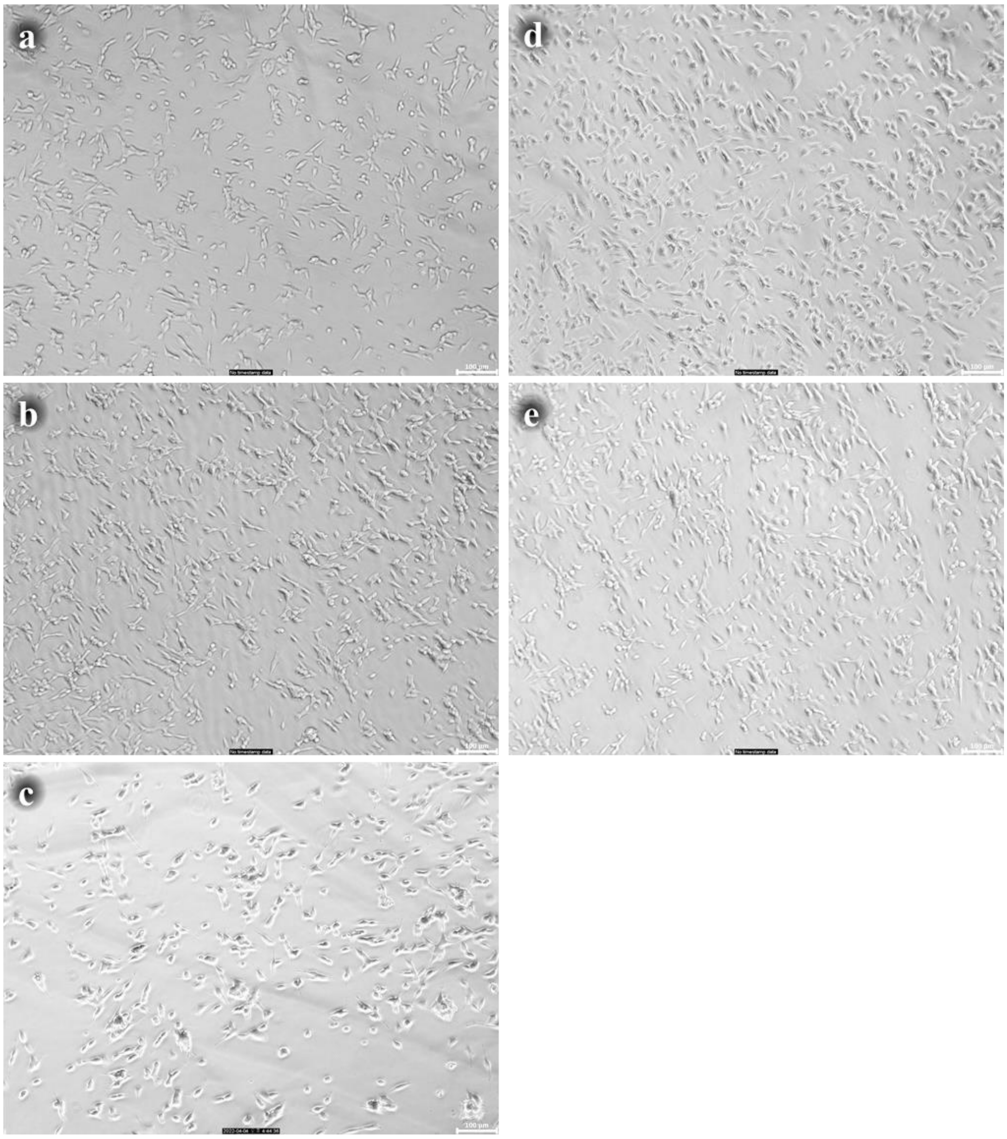

Figure S7: Representative brightfield images of the undifferentiated SH-SY5Y cells cultured in plates with different types of plate coatings.
Non-coated (a), 0.1% Gelatin B (wet) coated (b), 0.1% Gelatin B coated (c), 0.1 mg/mL Poly-L-Lysine (PLL) coated (d) and 0.1 mg/ml Polyethylenenamine (PEI) coated (e) plates were compared for their cell attachment capabilities. The coating was performed by adding 250 µL of aqueous coating solution per 4 cm2 of coat surface and subsequent 1-hour incubation in the cell-culture incubator. For wet surface Gelatin B coating (b), the plate surface was washed four times with sterilized water, and cells were directly added to the plate after washing. In all other cases (c, d, and e), plates were 1 hour dried in the cell-culture incubator upon washing three times and washed once prior to the cell plating. The cell attachment pattern was observed after 2-days. (10x, scale bar; 100 µm). The images were taken using a Leica phase contrast inverted microscope.

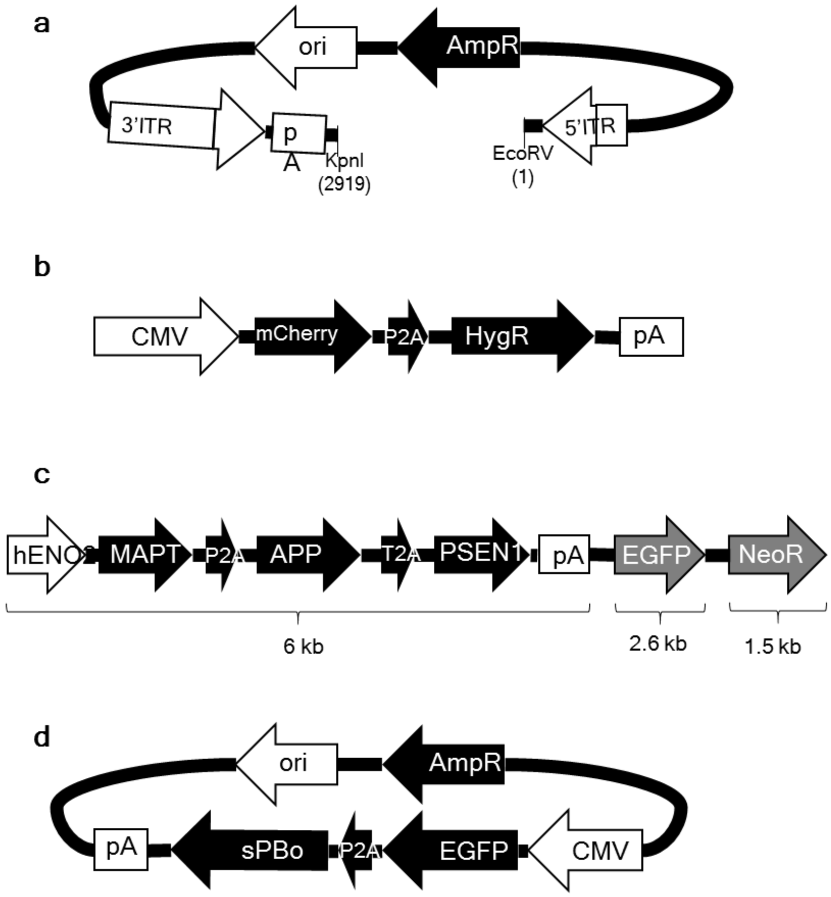

Figure S8: Vector maps.

All the vectors contain E. coli expressing the β-lactamase (AmpR) gene conferring ampicillin resistance and high-copy-number ColE1/pMB1/pBR322/pUC origin of replication (ori). a) Transposon backbone (2.9 kb) span from EcoRV to Kpn1 sites composed of TATA rich 5' ITR adjacent region, 35 bp piggyBac transposon-specific 5' inverted terminal repeat sequence (5' ITR), AmpR, ori, 63 bp 3' ITR, AATAAA rich 3' ITR adjacent region and, rabbit β-globin polyadenylation signal (pA). b) RFP cassette (2.5 kb), composed of human cytomegalovirus immediate early enhancer and early promoter (CMV) driven bi-cistronic ORF containing red fluorescent protein (mCyherry), P2A peptide from porcine teschovirus-1 polyprotein, hygromycin B phosphotransferase (HygR) and terminated by rabbit β-globin polyadenylation signal (pA). c) 3x(wt)AD-GFP construct composed of 6 kb expression cassette, 2.6 kb EGFP cassette, and 1.5 kb NeoR (aminoglycoside phosphotransferase) cassette. The human enolase 2 (ENO2/NSE) promoter-driven tricistronic expression cassette is composed of end-to-end knotted wild-type ORFs of human MAPT, APP, and PSEN1 genes separated by P2A and T2A (from *Thosea asigna virus* capsid protein) self-cleavage peptides. d) GFP-sPBo 5050 bp vector having human cytomegalovirus immediate early enhancer and early promoter (CMV) driven bi-cistronic ORF containing enhanced green fluorescent protein (EGFP), P2A, super piggyback transposase from transposase from Transposagen (Lonza) and terminated by rabbit β-globin polyadenylation signal (pA).

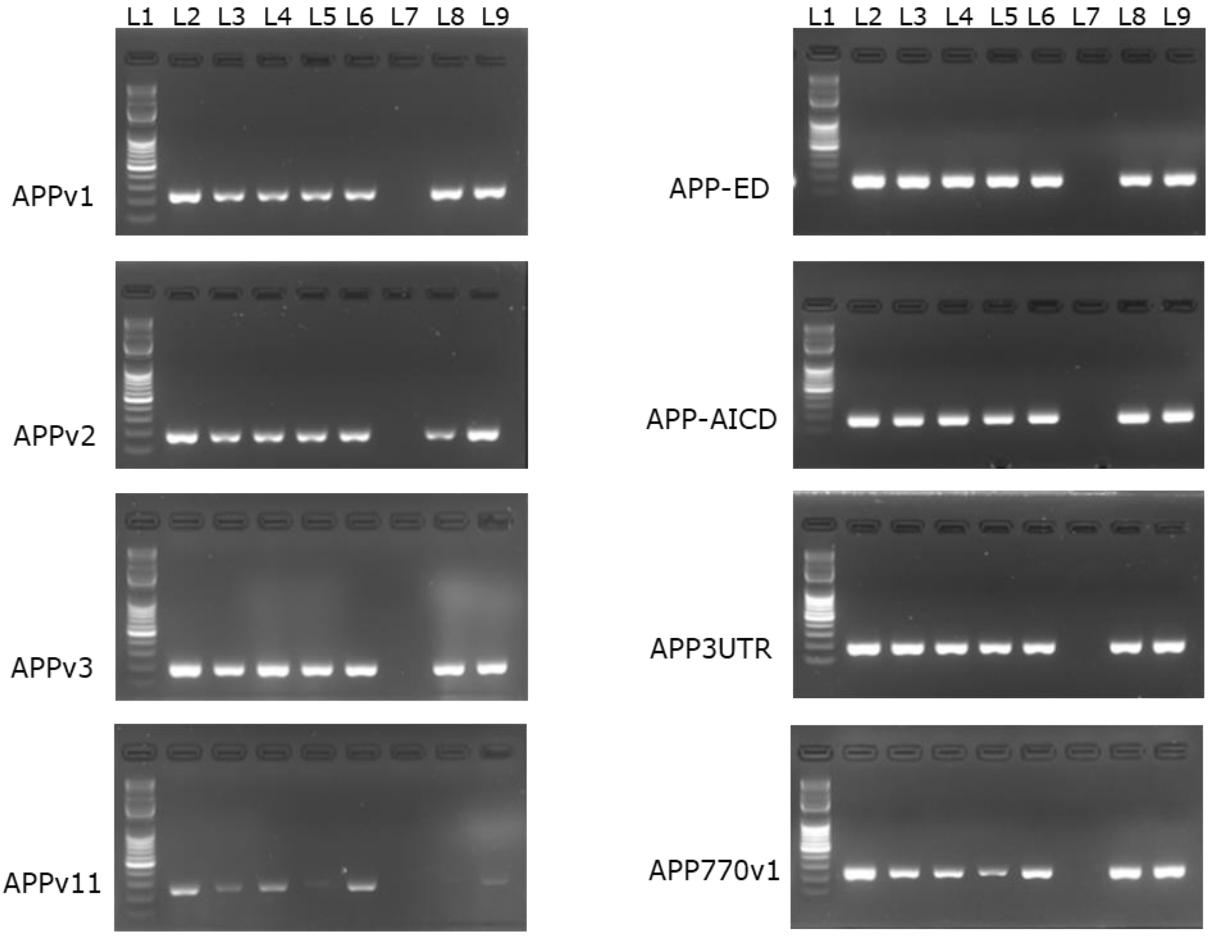

Figure S9: Full gel images of endogenous and transcript-group-targeted PCR of APP gene (Target info. in Table S3).

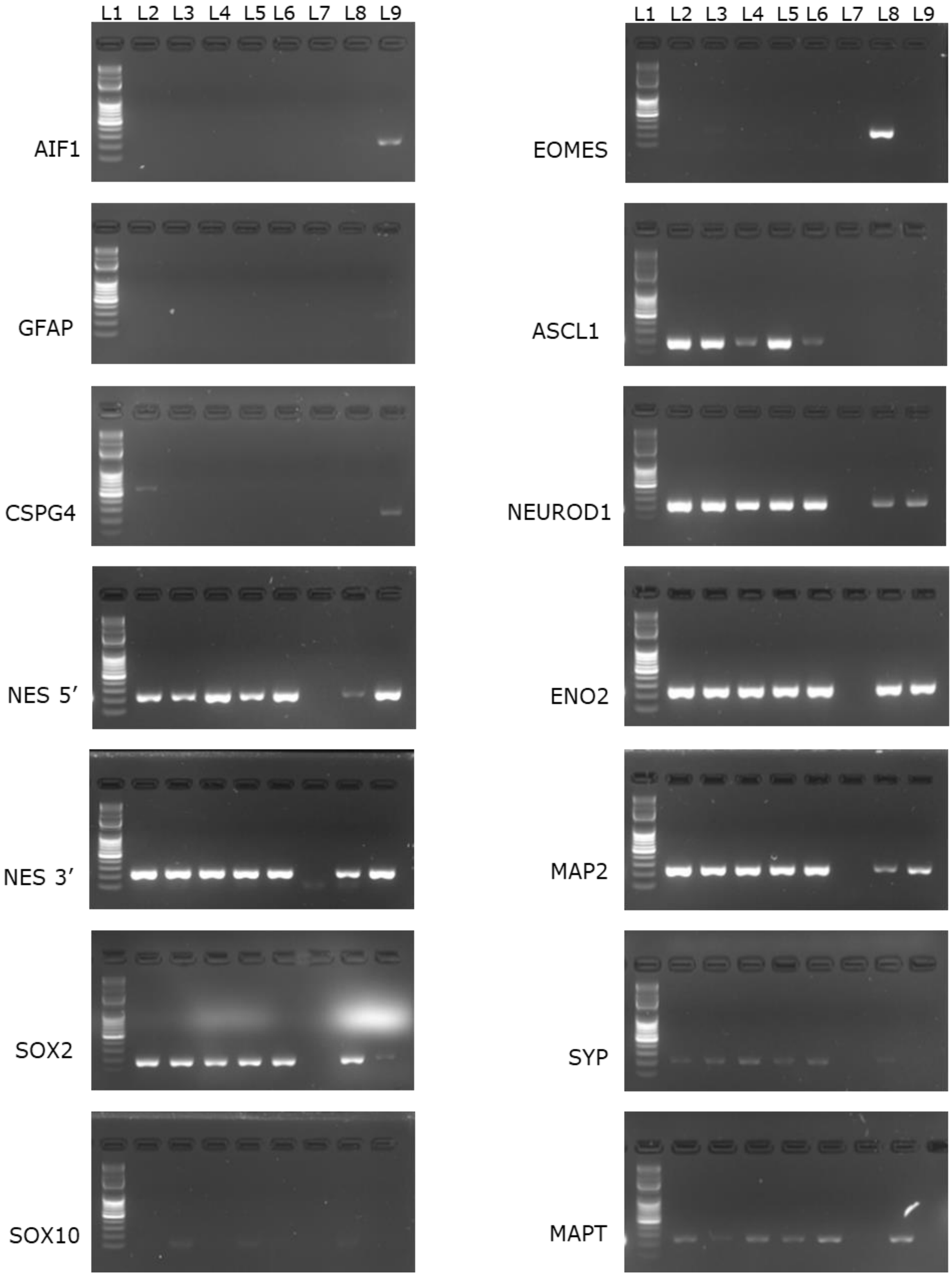

Figure S10: Full gel images of neuron differentiation state marker-targeted PCR (Target info. in Table S4).

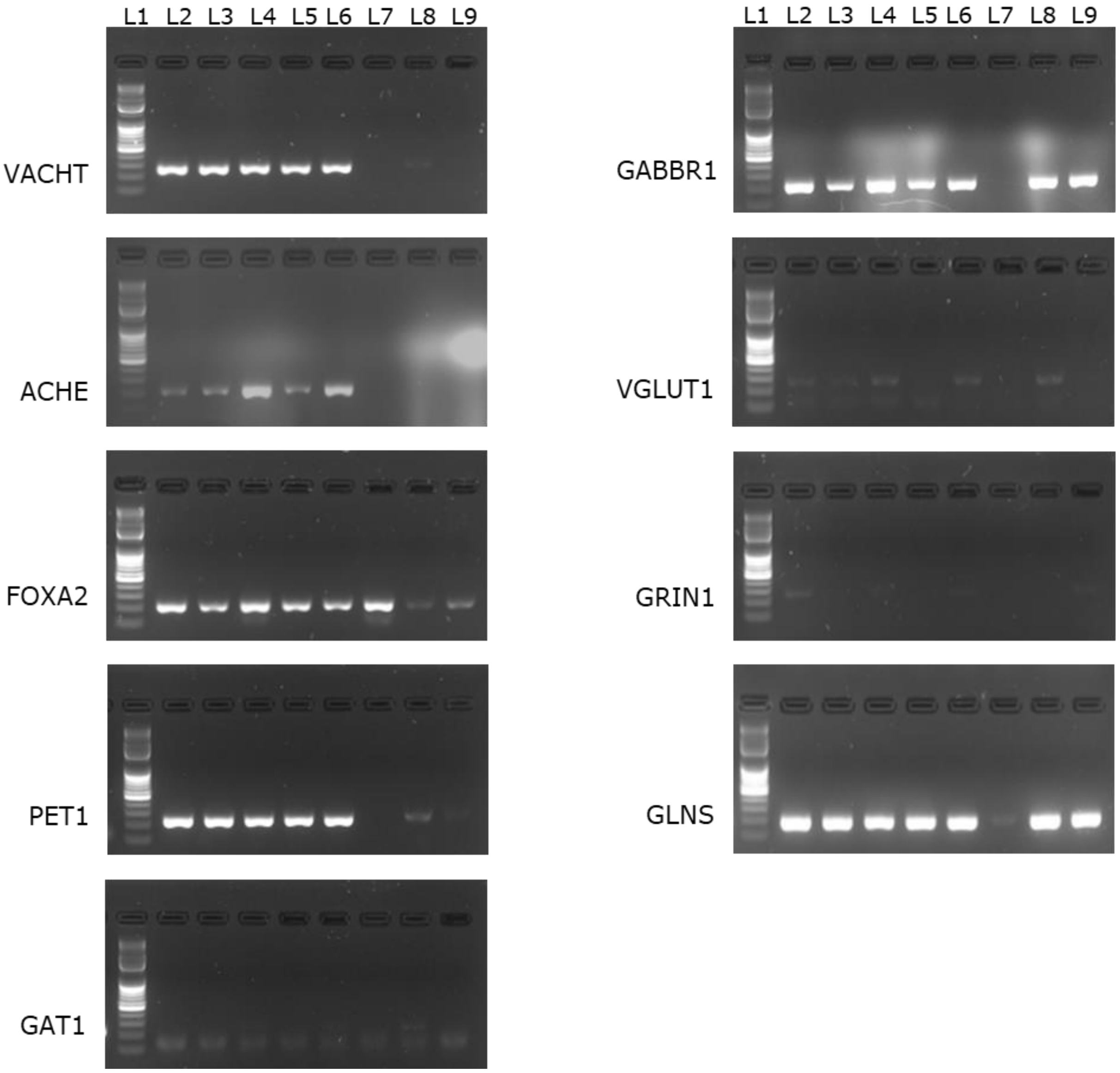

Figure S11: Full gel images of neurotransmitter marker-targeted PCR (Target info. in Table S5).

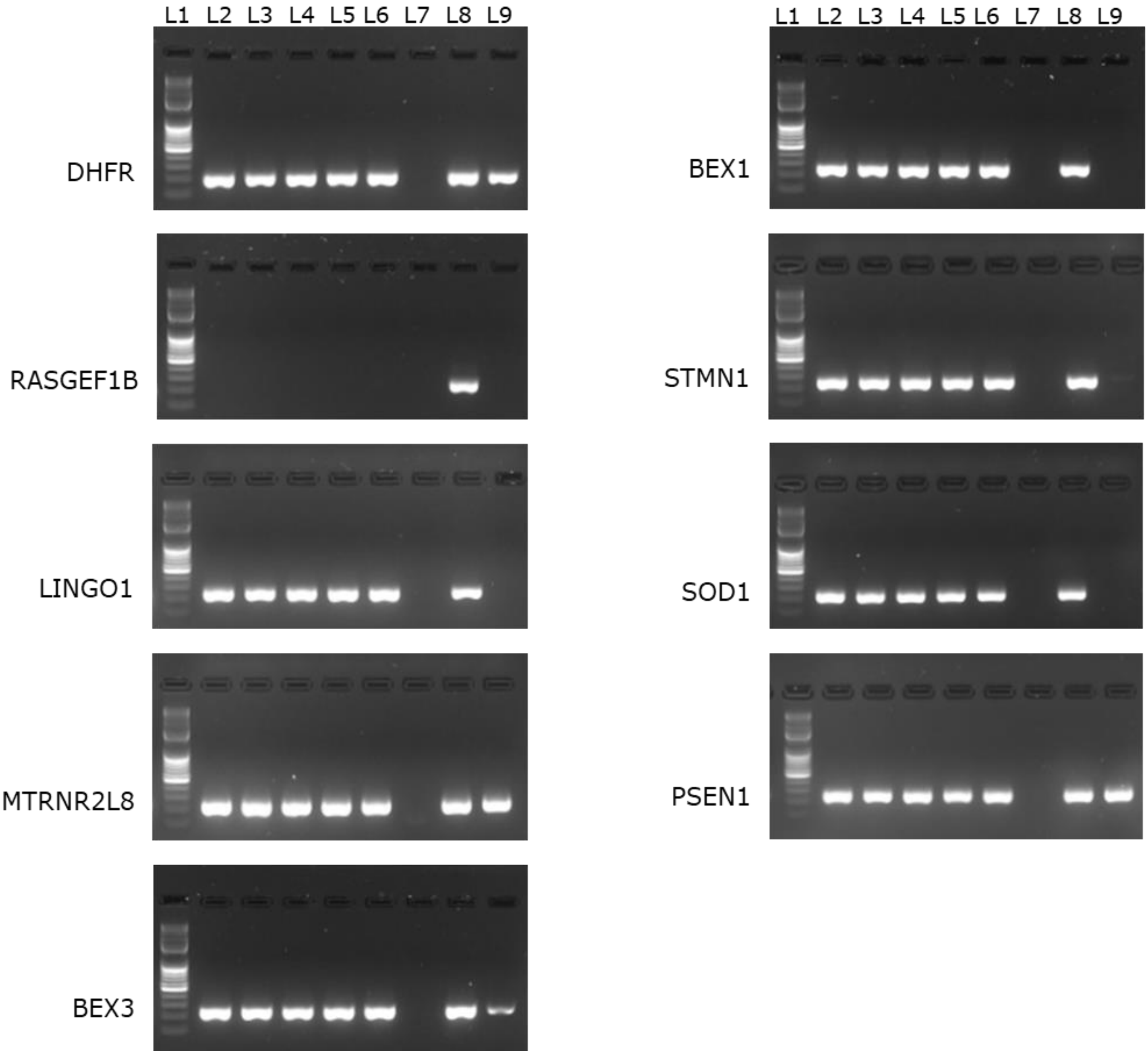

Figure S12: Full gel images of SH-SY5Y cell differentiation state responsive gene-targeted PCR (Target info. in Table S6).

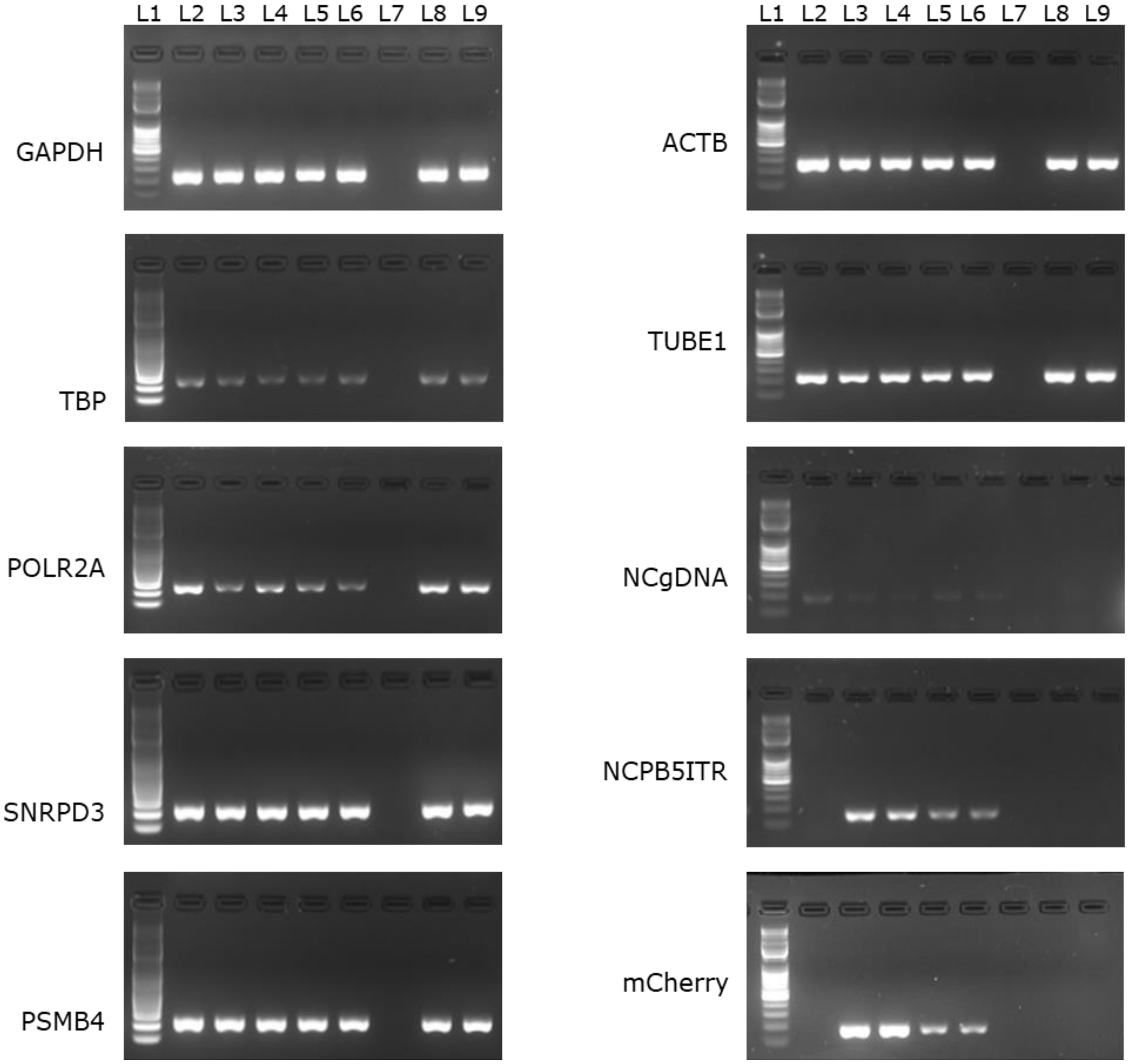

Figure S13: Full gel images of the housekeeping genes, gDNA, and transgenic markers used in PCR and RTQPCR (Target info. in Table S7).

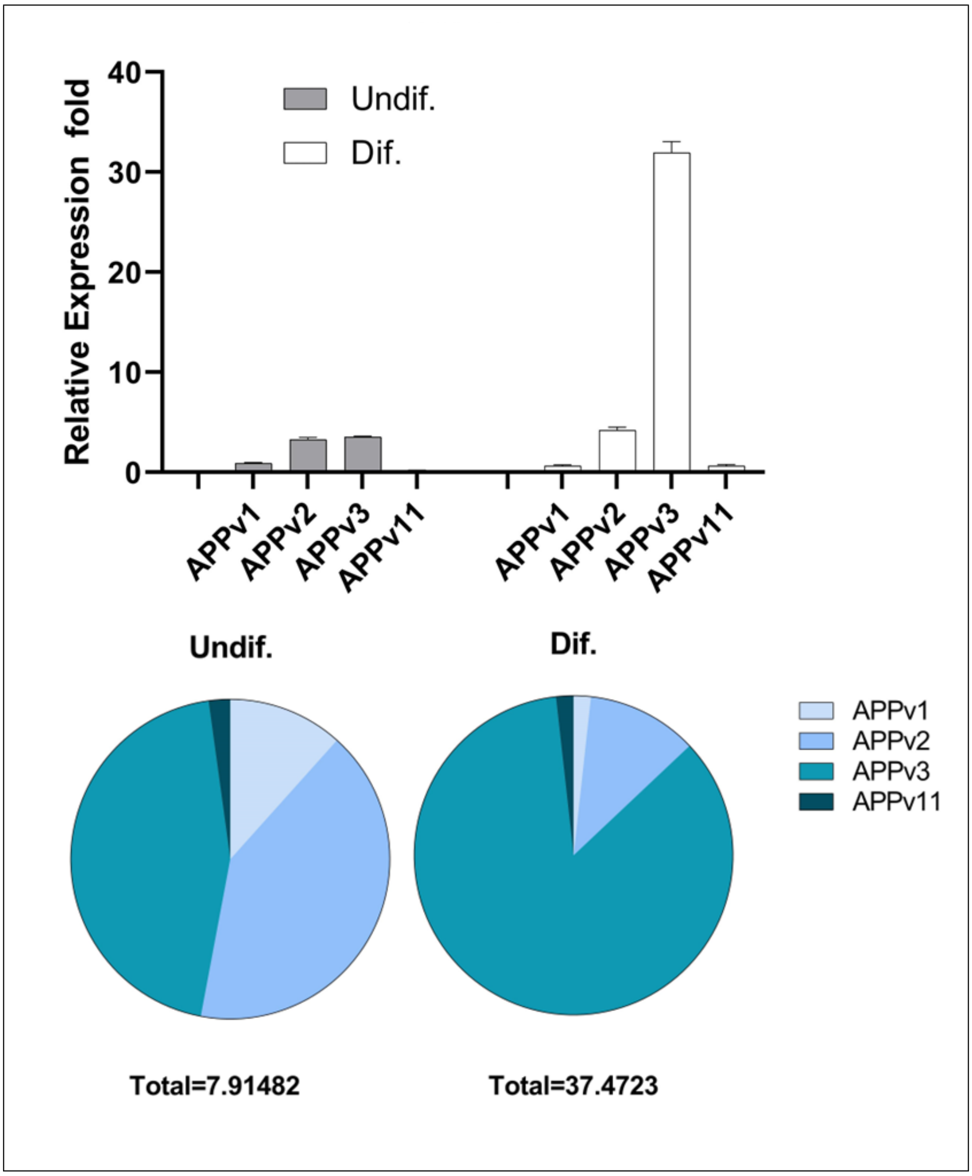

Figure S14: Quantitative comparison of APP transcript-variant dynamics among APPv1, APPv2, APPv3, and APPv11.

Relative transcript variant abundance of the immature (proliferative) SH-SY5Y cells in an undifferentiated state (Left) & the post-mitotic neuron-like SH-SY5Y cells at the *in-natura* terminal differentiated state (Right). The data presented in Figure 5a is reanalyzed here, normalized against the expression level of APPv1 in the undifferentiated cells, assuming equivalent qPCR efficiencies. The expression levels of APPv1, APPv2, APPv3, and APPv11 in the undifferentiated group are expressed as fold changes of 1.0, 3.5, 3.8, and 0.2, summing to 7.9, while in the differentiated group, the fold changes are 0.7, 4.6, 34.6, and 0.7, with a total of 37.5. The pie charts illustrate the relative contribution of each variant to the total expression within their respective differentiation states. Note the disproportionation of APPv1 (fallen) & APPv3 (risen) from left to right.

Supplementary Note

Although 3D neuronal cultures offer several advantages, the conventional 2D culture system provides a simpler and more cost-effective approach to culturing neurons. Additionally, we viewed it as a more user-friendly platform for data collection and imaging. Therefore, we adopted a 2D system in this experiment.

To enhance cell attachment, we sought a well-performing and affordable surface-coating material and chose Polyethyleneimine (PEI), a synthetic cationic polymer that is well-known as an efficient nucleic acid transfection agent.

PEI is a proven, effective surface coating agent in cell culture[1], and has shown evidence of its neuronal survival and differentiation potentials[2]. We tested PEI, along with two other low-cost plate-coating materials, gelatin and PLL, for their performance in cell attachment. PEI was chosen as the coating material since it resulted in a more uniformly dispersed, clump-free cell population. Serum deprivation arrests the cell cycle and triggers the differentiation of SH-SY5Y cells. Dramatic serum deprivation procedures have rendered cells increasingly stressed and vulnerable to death[3]. As described by Shipley et al. 2016[4], the serum content was reduced from 15% to 2.5% in the first serum deprivation step. Although we maintained the undifferentiated cells in a medium containing 15% serum, the addition of a two-day pre-acclimatization period, by reducing the serum by one-third (to 10%), allowed cells to stay stress-free and continue their growth on the differentiation surface. We further optimized the serum deprivation time-lapse by maintaining cells in 2.5% serum for four days, followed by 1%. Retinoic acid plays a vital role in the growth, differentiation, and survival of neurons, also enhancing the formation of connections between them and thus supporting neuronal plasticity[5]. A recent study suggested differentiation in the absence of cAMP and withdrawal of RA at a later stage of differentiation[6]. However, when performing differentiation without cAMP, we experienced a higher epithelial transformation tendency during the optimization steps (Fig. S15). Therefore, we used db-cAMP-amended media from the beginning of total serum discontinuation until terminal differentiation occurred. Furthermore, we continued RA supplementation throughout differentiation and into the post-differentiation period, considering the beneficial effects of RA on neurons[5]. A major concern of *in vitro* neural differentiation is the passaging or splitting of over-populated cells. Cell splitting causes damage and stresses in the neurons, which leads to ultimate cell loss[4]. We omitted the cell sub-culturing/splitting step by employing low-density seeding, maintaining the initial cell-seeding density at a very low level. This approach allowed cells to replicate without over-populating the differentiation surface, thus giving them enough time to acclimatize to the new surface.

Beginning with a smaller number of cells leads to a less dense cell population throughout the differentiation, which omits the sub-culturing requirement during the differentiation process. Avoiding passaging during differentiation reduces cell damage, facilitates better neurite elongation, and ultimately increases neuron survival. Moreover, this omission alleviates the burden in the differentiation procedure.

While 3D neuronal cultures have their own advantages, the conventional 2D culture system remains a simpler, cost-effective, and user-friendly approach for neuron culturing, data collection, and imaging. By adopting a 2D system, we achieved cell attachment using PEI, which proved to be an effective and affordable surface-coating material. PEI provided a uniformly dispersed, clump-free cell population, outperforming other tested materials such as gelatin or PLL. To mitigate stress and enhance differentiation, we optimized the serum

deprivation process and included a pre-acclimatization period. RA supplementation was maintained throughout the differentiation process, given its beneficial effects on neuronal growth and plasticity. We avoided cell sub-culturing by employing low-density seeding, which minimized cell damage, facilitated neurite elongation, and increased neuron survival. Our approach not only simplified the differentiation procedure but also improved the overall efficiency and reliability of the neuronal culture system.

Supplementary Tables

Protein length (AAs)	NCBI Ref. Seq.	Protein isoform	CDS length (Nts)	NCBI Ref. Seq.	mRNA Transc. Var.
770	NP_000475.1	a	2313	NM_000484.4	1
751	NP_958816.1	b	2256	NM_201413.3	2
695	NP_958817.1	c	2088	NM_201414.3	3
746	NP_001129488.1	d	2241	NM_001136016.3	4
639	NP_001129601.1	e	1920	NM_001136129.3	5
714	NP_001129602.1	f	2145	NM_001136130.3	6
660	NP_001129603.1	g	1983	NM_001136131.3	7
752	NP_001191230.1	h	2259	NM_001204301.2	8
733	NP_001191231.1	i	2202	NM_001204302.2	9
677	NP_001191232.1	j	2034	NM_001204303.2	10
714	NP_001372182.1	k	2145	NM_001385253.1	11

Table S1: Transcript variant information of APP gene. NLM/ NCBI reference sequence accession numbers of the APP mRNA transcript variants and corresponding protein isoforms.

Transcript variant and corresponding protein lengths (AAs) of each isoform

Variation of AA count in each isoform compared to major isoforms in each group

	**Group-1 (1, 6, 8)**	**Group-2 (2, 4, 9)**	**Group-3 (3, 5, 7, 10)**	**Group-4 (11)**
**0** (Major isoform)	1 [770]	2 [751]	3 [695]	11 [714]
**-5**		4 [746]		
**-18**	8 [752]	9 [733]	10 [677]	
**-35**			7 [660]	
**-56**	6 [714]		5 [639]	

Table S2: The isoform diversity of the APP gene.
APP isoforms were initially categorized into four groups based on the diversity of Exon 7 and 8 of each APP transcript variant, as mentioned in Table 1. The isoforms in each group were further organized by the size of their AA stretch (within "[]" s) and the secondary splice-site dependent variation of their AA count (0 to -56) compared to the corresponding major isoform.

Target site on APP transcript [NCBI RefSeq ID]	Purpose	bp	Name	Sequence (5'→3')	Size (Nt)	Tm°C Primer blast
**APP-ED** [All splice variants]	Total APP (native and transgenic)	147	qAPP-ED-F	CTGACCACTCGACCAGGTTCTGGG	24	66
			qAPP-ED-R	GTTTGAACCCACATCTTCTGCAAAGAACACC	31	66
**APP-AICD** [All splice variants]		143	qAPP-AICD-F	TGCTGAAGAAGAAACAGTACACATCCATTCATC	33	65
			qAPP-AICD-R	CTGCATCTGCTCAAAGAACTTGTAGGTTGG	30	66
**APP-3'UTR** [All splice variants]	Endogenous total APP	154	qAPP-3UTR2-F	CCTGATTATTTATCACATAGCCCCTTAGCCAG	32	65
			qAPP-3UTR2-R	GGAATACTTAGGCAAGAGAAGCAGCTGAAC	30	66
**APPv1.6.8Ex.7-8** [NM_000484.4] [NM_001136130.3] [NM_001204301.2]	Var. 1, 6 and 8 (Ex.7-8)	190	qAPP-770v1-F	CGAGCAATGATCTCCCGCTGGTAC	24	65
			qAPP-770v1-R	GTTTAACAGGATCTCGGGCAAGAGGTTC	28	65
**APPv1.6.8** [NM_000484.4] [NM_001136130.3] [NM_001204301.2]	.Var. 1, 6 and 8 (Ex.span)	199	qAPPv1.6.8-F(Ex7-8)	TGGCAGCGCCATGTCCCAAAG	21	66
			qAPPvAll-R(Ex9)	GGACATTCTCTCTCGGTGCTTGGC	24	65
**APPv2.4.9** [NM_201413.3] [NM_001136016.3] [NM_001204302.2]	Var. 2, 4 and 9 (Ex.span)	145	qAPPv2.4.9-F(Ex7-9)	GTGTGGCAGCGCCATTCCTACAAC	24	66
			qAPPvAll-R(Ex9)	GGACATTCTCTCTCGGTGCTTGGC	24	65
**APPv3.5.7.10** [NM_201414.3] [NM_001136129.3] [NM_001136131.3] [NM_001204303.2]	Var. 3, 5, 7 and 10 (Ex.span)	152	qAPPv3.5.7.10-F(Ex6-9)	CTGTGGAAGAGGTGGTTCGAGTTCCTAC	28	66
			qAPPvAll-R(Ex9)	GGACATTCTCTCTCGGTGCTTGGC	24	65
**APPv11** [NM_001385253.1]	Var. 11 (Ex.span)	204	qAPPv11-F(Ex6-8)	GAAGAGGTGGTTCGAGTGTCCCAAAG	26	65
			qAPPvAll-R(Ex9)	GGACATTCTCTCTCGGTGCTTGGC	24	65

Table S3: List of total, endogenous, and transcript-group specific primers targeted APP in PCR and RTQPCR (total nine primer pairs).

Differentiation state of Neurons	Target as, Gene Symbol|Aliase [NCBI RefSeq ID]	bp	Name	Sequence (5'→3')	Size (Nt)	Tm°C Primer blast
Non-neuronal/ Glial cells	**AIF1 | IBA1** [NM_032955.3]	177	qAIF1(IBA1)-F	GGAGTCCCCAAGACTCACCTAGAGC	25	65.6
			qAIF1(IBA1)-R	GCCTGTTGGCTTTTCCTTTTCTCTCGC	27	66.9
	**GFAP** [NM_002055.5]	157	qGFAP-F	CAAGAGGAACATCGTGGTGAAGACCG	26	65.5
			qGFAP-R	GGAGCAACTATCCTGCTTCTGCTCG	25	65.4
	**CSPG4 | NG2** [NM_001897.5]	201	qCSPG4(NG2)-F	CCACCCTTGCTGTGGCTGTGTC	22	66.1
			qCSPG4(NG2)-R	GGAACTGTGTGACCTGGAAGAGCAC	25	65.4
Neuroepithelial (Neural Stem)	**NES**5'**end** [NM_006617.2]	182	qNES(5end)-F	CTGGAGCAGGAGAAACAGGGCCTAC	25	66.6
			qNES(5end)-R	TGAAAGCTGAGGGAAGTCTTGGAGCC	26	66.5
	**NES3'end** [NM_006617.2]	178	qNES(3end)-F	CTGGGATGACAGCTTGAGGGGTG	23	65
			qNES(3end)-R	GCCTGCATCCTCCATCCCACTG	22	65.3
	**SOX2** [NM_003106.4]	151	qSOX2-F	CACCCACAGCAAATGACAGCTGC	23	65
			qSOX2-R	CACCCTCCCCAGGTTTTCTCTGTAC	25	64.5
	**SOX10** [NM_006941.4]	140	qSOX10-F	CCATCCAGGCCCACTACAAGAGC	23	65
			qSOX10-R	CTCTGTCTTCGGGGTGGTTGGAG	23	64.6
Intermediate progenitor	**EOMES | TBR2** [NM_001278182.2]	178	qEOMES(TBR2)-F	CTGGTTCCCACTGGATGAGACAGGAG	26	66.1
			qEOMES(TBR2)-R	GAGGGCTCATTCAAGTCCTCCACG	24	65
	**ASCL1 | MASH1** [NM_004316.4]	165	qASCL1(MASH1)-F	GAGGGCTCTTACGACCCGCTCAG	23	66.5
			qASCL1(MASH1)-R	CTCCCAACGCCACTGACAAGAAAGC	25	66.3
young neuron	**NEUROD1** [NM_002500.5]	207	qNEUROD1-F	CTCTTTCAAACACGAACCGTCCGC	24	64.8
			qNEUROD1-R	GCATTGAGCTGGGCACTCATGACTC	25	66
Mature Neurons	**ENO2 | NSE** [NM_001975.3]	209	qENO2(NSE)-F	GCACAGGCCAGATCAAGACTGGTG	24	65.7
			qENO2(NSE)-R	CACAGATCAGGACAGCAAGGTTCCAG	26	65.3
	**MAP2** [NM_001375553.1]	206	qMAP2-F	GTAACCAAGAGCCCAGAAAAGCGCTC	26	66.2
			qMAP2-R	GTAGACCCAGGGGTAGTGGGTGTTG	25	66
	**SYP** [NM_003179.3]	159	qSYP-F	GACTATGGGCAGCAAGGCTACGG	23	65.3
			qSYP-R	GAGGGGTGGAGACCTAGGGTATAGG	25	64.3
	**MAPT | TAU** [NM_016835.5]	196	qMAPT(Tau)-F	CAAGGACAGAGTCCAGTCGAAGATTGG	27	64.9
			qMAPT(Tau)-R	GACATTGCTGAGATGCCGTGGAGAC	25	65.8

Table S4: List of neuron differentiation state marker genes and corresponding primers used in PCR and RTQPCR (total 17 primer pairs).

Target as Gene Symbol|Aliase [NCBI RefSeq ID]	bp	Name	Sequence (5'→3')	Size (Nt)	Tm°C Primer blast
**SLC18A3|VACHT** [NM_003055.3]	206	qChol.SLC18A3(VACHT)-F	CCTCTGTTTTGGCATAGCCCTAGTCG	26	65.2
		qChol.SLC18A3(VACHT)-R	CCAGTCCCATGCCAAGGCTGAG	22	65.4
**ACHE** [NM_000665.5]	188	qChol.ACHE-F	CTCAGCAGTACGTTAGTCTGGACCTG	26	64.4
		qChol.ACHE-R	GTTCTTCCAGTGCACCATGTAGGAGC	26	65.6
**FOXA2|HNF3B** [NM_021784.5]	169	qDop.FOXA2-F	CAACAACCTCATGTCCTCGGAGCAG	25	65.5
		qDop.FOXA2-R	GTCCAGGCCCGTTTTGTTCGTGAC	24	66.5
**FEV|PET-1** [NM_017521.3]	173	qSer.FEV(PET1)-F	GGACCGAAGCTCCCTCAATCCTTG	24	65
		qSer.FEV(PET1)-R	GCGGAAATGCACTGGAGTGGTGG	23	66.4
**SLC6A1|GAT1** [NM_003042.4]	185	qGab.SLC6A1(GAT1)-F	GGTACATGGCCTACATGTTCCTCACC	26	65
		qGab.SLC6A1(GAT1)-R	GTGAGAGTGTCGGGTCGGTGAGTG	24	66.6
**GABBR1|GABABR1** [NM_001470.4]	186	qGab.GABBR1-F	CTGATCACCCGAGGGGAATGGC	22	65
		qGab.GABBR1-R	CTGCTGCCGAGACTGGAGTTGATG	24	65.5
		qGab.GAD2(GAD65)-R	GGGTTTGAGATGACCATGCGGAAG	24	64
**SLC17A7|VGLUT1** [NM_020309.4]	206	qGlut.SLC17A7(VGLUT1)-F	CATGACTAAGCACAAGACTCGGGAGG	26	64.9
		qGlut.SLC17A7(VGLUT1)-R	CATCCTCCATTTCGCTGTCGTCACTG	26	65.8
**GRIN1|NMDAR1** [NM_007327.4]	197	qGlut.GRIN1(NMDAR1)-F	CCTACAAGCGGCACAAGGATGCTC	24	66
		qGlut.GRIN1(NMDAR1)-R	GCTCGTGTCTTTGGAGGACCTACG	24	65
**GLUL|GLNS** [NM_001033044.4]	154	qGlut.GLUL(GLNS)-F	CAGGCTGCCATACCAACTTCAGC	23	64.1
		qGlut.GLUL(GLNS)-R	GTTAGACGTCGGGCATTGTCCAGG	24	65.5

Table S5: List of Neurotransmitter marker genes and corresponding primers used in PCR and RTQPCR (total 15 primer pairs).

Target as,<br>Gene Symbol|Aliase<br>[NCBI RefSeq ID]	bp	Name	Sequence (5'→3')	Size (Nt)	Primer-blast Tm°C
**MTRNR2L8**<br>[NM_001193596.3]**	174	qMTRNR2L8-F	GCATTCTCAGTATTAGAGGCACCGCC	26	65.5
		qMTRNR2L8-R	CACGGGCAGGTCAATTTCACTGG	23	64.3
**BEX3**<br>[NM_206917.3]	201	qBEX3-F	ACCAGCCTGCAGGAAATCGACG	22	65.4
		qBEX3-R	GCTCCCCCATAAGGATACGCAGAC	24	64.7
**BEX2|BEX1**<br>[NM_018476.4]	211	qBEX1-F	GCCCATCCTGCAGTATAGATGGGATATG	28	64.5
		qBEX1-R	GATTCAGGGCATAAGGCAAAACTCATCAT	29	64.1
**STMN1|SMN**<br>[NM_005563.4]	187	qSTMN1-F	GCTGCAGAAGAAAGACGCAAGTCCC	25	66.4
		qSTMN1-R	CAGCCATTTGTGCCTCTCGGTTCTC	25	66
**PSEN1|PS1**<br>[NM_000021.4]	207	qPSEN1(PS1)-F	GTTCAGTGAGGAATGGGAAGCCCAG	25	65.3
		qPSEN1(PS1)-R	CAGTCTCCACTGGCTGTTGCTGAG	24	65.4
**DHFR|DHFR1**<br>[NM_000791.4]	156	qDHFR-F	GAAGCCATGAATCACCCAGGCCATC	25	65.9
		qDHFR-R	GCCTTTCTCCTCCTGGACATCAGAG	25	64.4
**RASGEF1B|GPIG4**<br>[NM_152545.3]	192	qRASGEF1B-F	TTTATGACATGGAAACAAGTGGAGTGTCC	29	63.7
		qRASGEF1B-R	GTGTTAAACTCTGCCTAAGAGGCTCGAC	28	65.2
**LINGO1|MRT64**<br>[NM_032808.7]	184	qLINGO1-F	CCACTGTGCCTTTCCCCTTCGAC	23	65.6
		qLINGO1-R	CTGATGCCTGCGTCCGACTTTCG	23	66.2
**SOD1**<br>[NM_000454.5]	163	qSOD1-F	CCATTGCATCATTGGCCGCACAC	23	65.7
		qSOD1-R	GGCCTCAGACTACATCCAAGGGAATG	26	64.8

Table S6: List of AD-responsive genes described by Mathys et al (2019) used in PCR and RTQPCR (total nine primer pairs).

Description			Target as, Gene Symbol|Aliase [NCBI RefSeq ID]	bp	Name	Sequence (5'→3')	Size (Nt)	Primer-blast Tm°C
Housekeeping genes	Energy Metabolism		**GAPDH|G3PD** [NM_002046.7]	186	qGAPDH-F	GCTGAGAACGGGAAGCTTGTCATCAATG	28	66
					qGAPDH-R	GGCAGAGATGATGACCCTTTTGGCTC	26	65
	Transcription		**TBP|TFIID** [NM_003194.5]	194	TBP-F	CAGAATATGGTGGGGAGCTGTGATGTGAAG	30	67
					TBP-R	TCTGCTCTGACTTTAGCACCTGTTAATACAAC	32	65
			**POLR2A** [NM_000937.5]	158	POLR2A-F	GCCGCACACGGTGAGAGTGAC	21	66
					POLR2A-R	CCAAAGAACATGCCGGTGGGTCC	23	66
	RNA processing		**SNRPD3|SMD3** [NM_004175.5]	192	SNRPD3-F	GAGATGGCCGAGTGGCACAGC	21	66
					SNRPD3-R	CACGTCCTCTTCCTCTTGCGGC	22	66
	Proteasome component		**PSMB4** [NM_002796.3]	167	PSMB4-F	CCGGCGCTCGAAGATGAACCC	21	65
					PSMB4-R	GGTCTGGCTTAGCACTGGCTGC	22	67
	Structural	Cytoskeleton	**ACTB|β-actin** [NM_001101.5]	209	qACTB-F	GAGCACAGAGCCTCGCCTTTGC	22	66
					qACTB-R	CTTCTGACCCATGCCCACCATCAC	24	65
		centriole	**TUBE1|TUBE** [NM_016262.5]	169	TUBE1-F	GCTTCGGGCAGACCCCAAACAC	22	66
					TUBE1-R	AGGAGGTACGGAACACAGGCTGG	23	66
mCherry reporter gene expressed in Mock/RFP cells			**mCherry** [*]	135	qmCherry-F	CCACTACGACGCTGAGGTCAAGAC	24	65
					qmCherry-R	CGTTCGTACTGTTCCACGATGGTGTAG	27	65
piggyBac 5'end transposon-specific inverted terminal repeat (5'ITR), Vector detection (negative control)			**NCPB5ITR** [*]	118	qNCPB5ITR-F	CGTCATTTTGACTCACGCGGTCG	23	66
					qNCPB5ITR-R	CCGTCGCTGTGCATTTAGGACATCTC	26	65
Un-transcribed region of chromosome 21, GRCh38.p13 Primary Assembly. APP promoter region, 1Kb upstream from the transcription start site, gDNA contamination marker (negative control)			**NCgDNA** [NC_000021.9]	159	qNCgDNA-F	TGACTTCCCAGGAAGGAAGTCTGTACC	27	65
					qNCgDNA-R	GGCTTGGAGCGAAGGACTGAAGC	23	66

Table S7: List of housekeeping genes, gDNA, and transgenic expression markers used in PCR and RTQPCR (total ten primer pairs). The vector components are denoted with "*."

References

[1] Vancha, A. R. et al. Use of polyethyleneimine polymer in cell culture as attachment factor and lipofection enhancer. BMC Biotechnol. 4, 23 (2004).
[2] Bledi, Y., Domb, A. J. and Linial, M. Culturing neuronal cells on surfaces coated by a novel polyethyleneimine-based polymer. Brain Res. Protoc. 5, 282–289 (2000).
[3] Macleod, M. R. et al. Serum withdrawal causes apoptosis in SH-SY5Y cells. Brain Res. 889, 308–315 (2001).
[4] Shipley, M. M., Mangold, C. A. and Szpara, M. L. Differentiation of the SH-SY5Y Human Neuroblastoma Cell Line. J. Vis. Exp. (108), e53193 (2016).
[5] Jacobs, S. et al. Retinoic acid is required early during adult neurogenesis in the dentate gyrus. PNAS 103, 3902–3907 (2006).
[6] Dravid, A., Raos, B., Svirskis, D. and O'Carroll, S. J. Optimised techniques for high-throughput screening of differentiated SH-SY5Y cells and application for neurite outgrowth assays. Sci. Rep. 11, 23935 (2021).
